# H3K14la drives endothelial dysfunction in sepsis‐induced ARDS by promoting SLC40A1/transferrin‐mediated ferroptosis

**DOI:** 10.1002/mco2.70049

**Published:** 2025-01-14

**Authors:** Fangchen Gong, Xiangtao Zheng, Wen Xu, Rongli Xie, Wenbin Liu, Lei Pei, Ming Zhong, Wen Shi, Hongping Qu, Enqiang Mao, Zhitao Yang, Ranran Li, Erzhen Chen, Ying Chen

**Affiliations:** ^1^ Department of Emergency Ruijin Hospital Shanghai Jiao Tong University School of Medicine Shanghai P.R. China; ^2^ Department of Critical Care Medicine Ruijin Hospital Shanghai Jiao Tong University School of Medicine Shanghai P.R. China; ^3^ Department of General Surgery Ruijin Hospital Lu Wan Branch Shanghai Jiaotong University School of Medicine Shanghai P.R. China; ^4^ Shanghai Institute of Aviation Medicine Shanghai Jiao Tong University Medical School Affiliated Ruijin Hospital Shanghai P.R. China

**Keywords:** endothelial dysfunction, ferroptosis, lactylation, lung injury, sepsis

## Abstract

Pulmonary endothelial cell (EC) activation is a key factor in acute respiratory distress syndrome (ARDS). In sepsis, increased glycolysis leads to lactate buildup, which induces lysine lactylation (Kla) on histones and other proteins. However, the role of protein lactylation in EC dysfunction during sepsis‐induced ARDS remains unclear. Integrative lactylome and proteome analyses were performed to identify the global lactylome profile in the lung tissues of septic mice. Cut&Tag analysis was used to identify the transcriptional targets of histone H3 lysine 14 lactylation (H3K14la) in ECs. Septic mice presented elevated levels of lactate and H3K14la in lung tissues, particularly in pulmonary ECs. Suppressing glycolysis reduced both H3K14la and EC activation, suggesting a link between glycolysis and lactylation. Moreover, H3K14la was enriched at promoter regions of ferroptosis‐related genes such as transferrin receptor (TFRC) and solute carrier family 40 member 1 (SLC40A1), which contributed to EC activation and lung injury under septic conditions. For the first time, we reported the role of lactate‐dependent H3K14 lactylation in regulating EC ferroptosis to promote vascular dysfunction during sepsis‐induced lung injury. Our findings suggest that manipulation of the glycolysis/H3K14la/ferroptosis axis may provide novel therapeutic approaches for sepsis‐associated ARDS.

## Graphical abstract

During sepsis, lactate‐dependent H3K14 lactylation at the promoter regions of ferroptosis‐related genes (TFRC, SLC40A1) promoted ferroptosis in ECs, which drives vascular dysfunction in sepsis‐related ARDS.

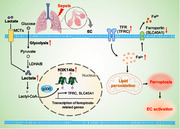

## 1 INTRODUCTION

Sepsis is a life‐threatening condition characterized by infection‐induced dysregulation of immune responses and multiple organ dysfunction.^1^ Acute respiratory distress syndrome (ARDS) is a devastating complication of sepsis, the development of which is associated with poor prognosis and increased mortality in patients with sepsis.^2^ Aberrant metabolism is increasingly recognized as a pivotal factor contributing to the pathogenesis of sepsis‐related organ dysfunction.^3^ Metabolic reprogramming favors aerobic glycolysis to meet the sepsis‐induced rapid demand for adenosine 5′‐triphosphate (ATP), which causes deregulated immune activation and severe tissue damage.^4^ Previous studies have indicated that glycolysis inhibition could improve outcomes in sepsis and sepsis‐related ARDS.^5^, ^6^ Elevated serum lactate levels, stemming from glycolysis, are positively correlated with the severity and mortality of sepsis and sepsis‐related organ dysfunction.^7^

Recently, lactate‐dependent lysine lactylation (Kla) has emerged as a novel form of post‐translational modification (PTM).^8^ Accumulating evidence has highlighted the critical regulatory role of Kla on core histones in various processes, including macrophage polarization, oncogenesis, neural excitation, and the pathogenesis of Alzheimer's disease.^9^, ^10^, ^11^, ^12^ Despite these advances, the specific regulatory role of lactylation in sepsis‐induced ARDS remains largely unexplored. Given the importance of glycolysis and lactate production in sepsis, understanding the role of lysine lactylation in this context is crucial for elucidating the molecular mechanisms of sepsis‐related organ damage.

Vascular endothelial cells (ECs), which comprise the inner surface of blood vessels, actively engage in the pathogenesis of sepsis‐induced ARDS.^13^ Upon the onset of sepsis, activated ECs orchestrate the infiltration of leukocytes by adhesion molecules such as vascular adhesion molecule‐1 (VCAM‐1), leading to lung injury via the release of proteases and oxygen‐derived radicals.^14^ In addition, the expression of tissue factor (TF) in activated ECs initiates intravascular coagulation and promotes platelet aggregation and microthrombi formation, aggravating microcirculatory disturbance and tissue injury in sepsis.^15^ Difficulties in recovery from EC injury lead to destruction of the blood‒air barrier, resulting in vascular leakage‐associated lung tissue edema and triggering ARDS.^16^ Under septic conditions, excessive glycolysis promotes the synthesis of macromolecules for EC activation and increases the massive production of lactate as the end‐product. EC‐specific inhibition of glycolysis has been reported to protect mice from acute lung injury in endotoxemia.^5^ The role of lactate‐derived lysine lactylation in EC activation during sepsis‐induced ARDS needs further evaluation.

In this study, we observed marked increases in global lysine lactylation and H3K14la levels in septic lung tissues as well as in sepsis‐induced ECs. Notably, the inhibition of H3K14la significantly ameliorated EC activation and lung injury under septic conditions. The enrichment of H3K14la at the promoter regions of ferroptosis‐related genes (TFRC and SLC40A1), promoted ferroptosis in ECs in response to LPS. Glycolytic inhibition significantly reduced EC ferroptosis by decreasing the level of H3K14la. Additionally, sepsis‐induced pulmonary microvascular inflammation and lung injury were ameliorated upon the blockade of ferroptosis. Our findings emphasize the pivotal roles of glycolysis‐mediated H3K14 lactylation and the subsequent induction of ferroptosis in EC activation and lung injury, providing promising therapeutic avenues for the treatment of sepsis‐related ARDS.

## 2 RESULTS

### 2.1 Protein lactylation was induced in sepsis‐related lung injury

**FIGURE 1:**
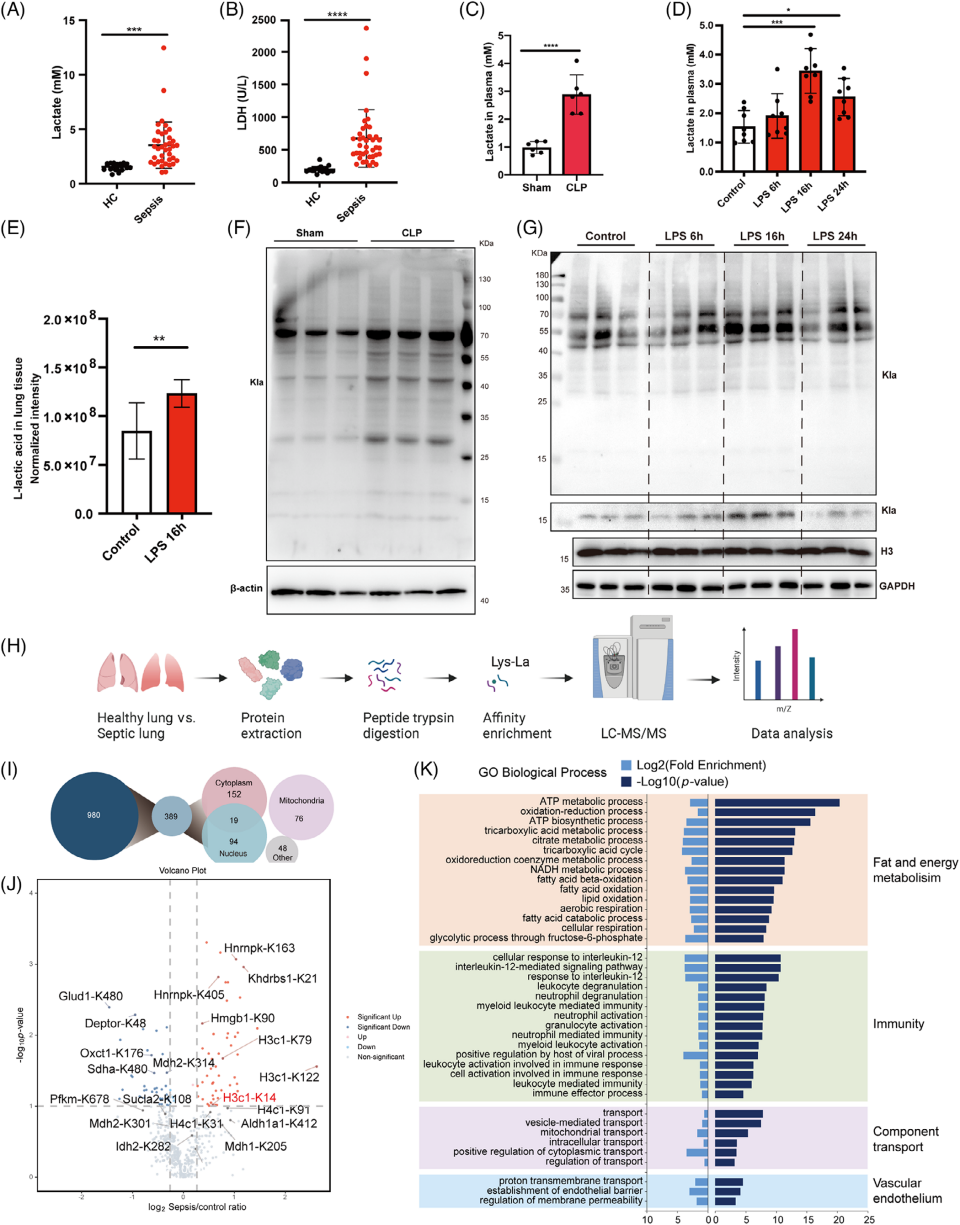
Global lysine lactylation was induced in sepsis‐induced lung injury. (A, B) Serum lactate and lactate dehydrogenase (LDH) levels in healthy controls (*n* = 20) and patients with sepsis (*n* = 38) were measured. (C) Plasma lactate levels were measured in the cecal ligation and puncture (CLP)‐induced sepsis model (*n* = 6 mice per group). (D, E) Mice were intraperitoneally (i.p.) injected with LPS (5 mg/kg). Saline (0.9% NaCl) was i.p. injected as a vehicle control. Lactate levels in the plasma were measured (*n* = 8 mice per group). The levels of L‐lactic acid in the lung tissues of the mice were obtained by from the metabolomics analysis (*n* = 9 mice per group). (F) Immunoblot analysis of global lysine lactylation in lung tissues from the CLP and sham groups. Histone H3 was used as the loading control. (G) Immunoblot analysis of global lysine lactylation in lung tissues from septic mice after LPS challenge at the indicated times. Histone H3 was used as the loading control. (H) Flow diagram of the liquid chromatography‐tandem mass spectrometry (LC‒MS/MS) analysis of lactylated proteins in lung tissues from septic and control mice. (I) Subcellular localization of lysine lactylated sites and proteins. (J) GO analysis of lysine lactylated proteins in lung tissues from the two groups of mice. (K) Volcano plot of the significantly changed lactylated proteins in lung tissues.

Compared with those in healthy controls, significantly elevated serum levels of lactate and lactate dehydrogenase (LDH) were observed in patients with sepsis (Figure 1A,B). Consistent with the elevated lactate levels in the plasma and lung tissues of septic mice (Figure 1C–E), increased expression of lysine lactylation (Kla) was observed in the lung tissues of septic mice (Figures 1F,G, and S1A,S1B).

To explore the possible role of protein lactylation in sepsis‐related ARDS, integrative lactylome and proteome analyses were performed using a label‐free quantification approach to characterize the lactylomes in the lung tissues of septic and control mice (Figure 1H). A total of 980 Kla sites across 389 proteins were identified from septic and control mice. The 389 lysine‐lactylated proteins were further classified according to their subcellular localization. In total, 171, 113, and 76 lysine‐lactylated proteins were located in the cytoplasm, nuclei, and mitochondria, respectively (Figure 1I). Motif analysis revealed the sequence characteristics of the 10 upstream and downstream amino acids of lysine (K), indicating the frequencies of different amino acids close to the lactylated lysine (Figure S1C). Gene Ontology (GO) analysis revealed that the lysine‐lactylated proteins were enriched mainly in biological processes associated with fat and energy metabolism, immune responses, component transport, and vascular endothelium (Figure 1J), suggesting the diverse biological functions of lactylated proteins in sepsis‐related lung injury. Additionally, among all these lactylated proteins, several inflammation‐related and cytoskeleton‐related proteins were identified (Figure S1D,E). Lactylated proteins associated with metabolism exhibited cross interactions (Figure S1F). A quantitative comparison of the lactylome between septic and control lung tissues revealed both up‐ and downregulated Kla sites (Figure 1K). Among others, 67 proteins with 86 Kla sites were significantly different between the two groups, of which 49 Kla sites were upregulated, and 37 Kla sites were downregulated in septic lung tissues compared with those in the control group (Table S1). These data indicate that sepsis‐induced global lysine lactylation in lung tissues may participate in modulating extensive biological functions.

### 2.2 H3K14 lactylation promoted vascular dysfunction in sepsis‐related lung injury

**FIGURE 2:**
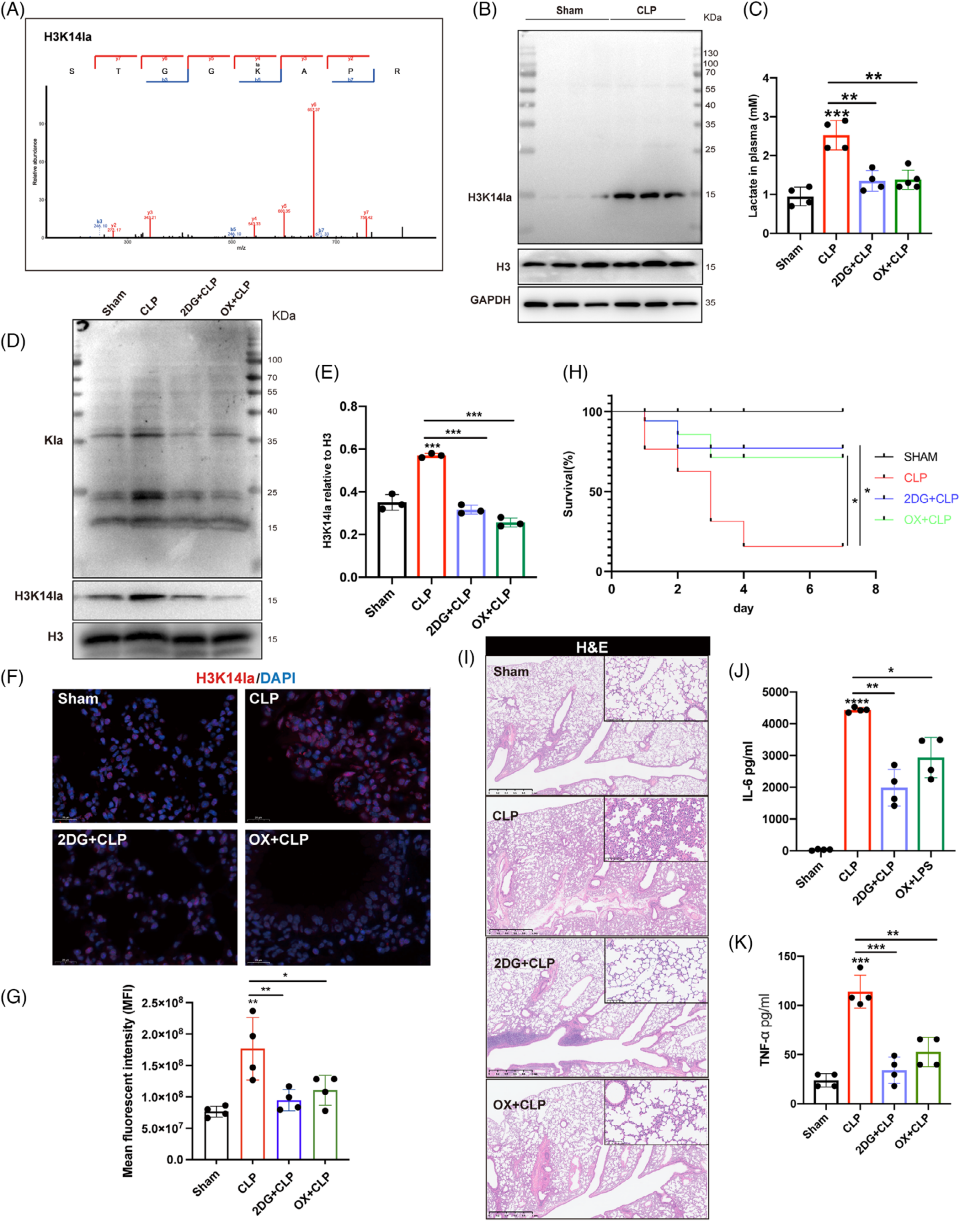
Inhibition of lactate production alleviated sepsis‐induced lung injury. (A) Tandem mass spectrometry (MS/MS) spectra of a lactylated histone peptide (H3K14la). (B) H3K14la expression levels were detected in lung tissues from the cecal ligation and puncture (CLP) and sham groups by immunoblotting. Histone H3 was used as the loading control. (C) Mice were administered 2DG (250 mg/kg) or sodium oxamate (OX, 500 mg/kg) 6 h prior to CLP or sham surgery. The plasma levels of lactate in the mice were measured (n = 4). (D, E) Immunoblot analysis of global lysine lactylation and H3K14la in lung tissues from the sham, CLP, 2DG+CLP, and OX+CLP groups (*n* = 3). (F, G) Immunofluorescence staining of H3K14la in lung tissues from the different groups. (H) The survival rate was compared among sham group, CLP group, 2DG+CLP group, OX+CLP group by Kaplan–Meier test (*n* = 15 for each group). (I) Lung injury was evaluated by hematoxylin‒eosin (H&E) staining. (J, K) The plasma levels of interleukin‐6 (IL‐6) and tumor necrosis factor alpha (TNFα) were measured by ELISA (*n *= 4).

Post‐translational modifications of histone proteins play important roles in modulating the global gene transcription. In the lactylated proteins, the MS/MS spectra of a lactylated histone peptide for H3K14la derived from the lung tissue revealed the occurrence of lactylation modification on H3K14 (Figure 2A). Immunoblots using a specific antibody against H3K14la (the antibody specificity is validated in Figure S2A) confirmed the increase in H3K14la in the lung tissue of septic mice (Figure 2B, Figure S2B). These data suggest that H3K14la may mediate the regulation of sepsis‐induced lung injury by lactate.

To investigate the impacts of lactate‐dependent H3K14 lactylation on sepsis‐induced lung injury, the nonmetabolizable glucose analog 2‐deoxy‐D‐glucose (2DG), a glycolytic inhibitor, and oxamate, an LDH inhibitor were used to inhibit lactate production in vivo (Figure 2C). In the lung tissues of septic mice, 2DG and oxamate administration significantly reduced the levels of histone Kla and H3K14la (Figure 2D,E; Figure S2C,D). Additionally, 2DG and oxamate dramatically improved the survival rate of septic mice (Figure 2H). 2DG and oxamate also mitigated sepsis‐induced lung tissue injury as well as systemic proinflammatory responses as represented by decreased plasma levels of cytokine IL‐6 and TNFα (Figure 2I–K; Figure S2E,F).

**FIGURE 3:**
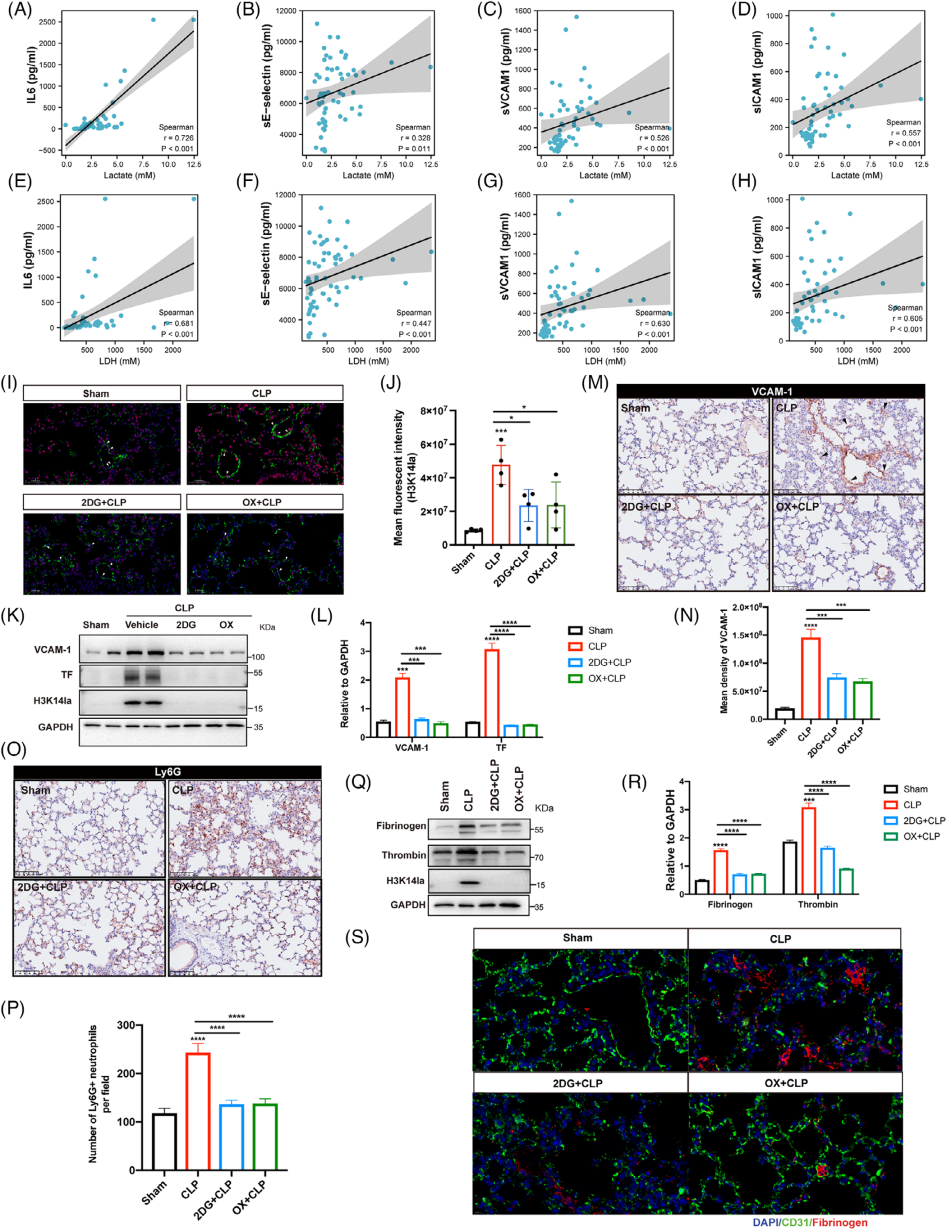
H3K14la was associated with endothelial cell (EC) activation in sepsis‐induced lung injury. (A–H) Correlations between serum lactate, lactate dehydrogenase (LDH), interleukin‐6 (IL6), soluble E‐selectin (sE‐selectin), sICAM1, and sVCAM1 among healthy controls and patients with sepsis were measured (healthy controls: *n* = 20; patients with sepsis: *n* = 38). (I, J) Immunofluorescence staining of H3K14la in the lung tissues of CLP and sham control mice. H3K14la is stained red; the pulmonary EC marker CD31 is stained green; and the nuclei are stained blue. The white arrowheads indicate H3K14la expressed in pulmonary ECs. (K, L) Immunoblot analysis of the protein expression of VCAM‐1 and TF in the lung tissues of the CLP and sham groups. (M, N) The expression and localization of VCAM‐1 in lung tissues from the CLP and sham groups were detected by immunohistochemical (IHC) staining. (O, P) IHC staining of neutrophil (Ly6G+) infiltration in mouse lung tissues from the CLP and sham groups. (Q, R) Immunoblot analysis of the expression of fibrinogen in the lung tissues of the CLP and sham groups. (S) Immunofluorescence staining of fibrinogen in the lung tissues of CLP and sham control mice. Fibrinogen is stained red; the pulmonary EC marker CD31 is stained green; and the nuclei are stained blue.

Given the regulatory role of H3K14 modification in EC functions, we further identified the participation of lactate‐derived H3K14 lactylation in regulating EC activation during sepsis related lung injury.^17^, ^18^ We first evaluated the serum levels of lactate, LDH, the inflammatory cytokine IL6, and the EC activation‐related molecules soluble E‐selectin (sE‐selectin), sICAM1, and sVCAM1, as well as their correlations in patients with sepsis. Lactate and LDH were positively correlated with IL6, sE‐selectin, sICAM1, and sVCAM1 (Figure 3A–H), suggesting a possible association between lactate production and EC activation and the inflammatory response during sepsis. Immunofluorescence costaining was then performed for H3K14la and the EC‐specific marker CD31 to determine the levels of H3K14la in the pulmonary microvasculature. The colocalization of H3K14la with CD31 provided evidence of increased H3K14la in pulmonary ECs in septic mice (Figure 3I,J). When lactate and H3K14la were decreased by 2DG and oxamate, H3K14la was downregulated (Figure 3I,J). Consistent with the increased H3K14la levels, expression levels of both VCAM‐1 and TF, two biomarkers of EC activation, were decreased by 2DG and oxamate pretreatment, indicating the alleviation of sepsis‐induced pulmonary EC activation (Figure 3K–N; Figure S2G–J). Increased expression of adhesion molecules plays a critical role in mediating sepsis‐induced infiltration of immune cells into underlying tissues.^19^ The results showed that 2DG and oxamate inhibited the infiltration of neutrophils into the lung tissues of septic mice (Figure 3O,P; Figure S2K,L). Fibrinogen is a thrombotic risk factor that converts fibrinogen to fibrin, which is the insoluble protein end‐product of blood coagulation.^20^ Our results revealed that the increased levels of fibrinogen in septic lung tissues were effectively reduced by 2DG and oxamate administration (Figure 3Q–S; Figure S2M–P). These data suggest that lactate‐induced H3K14la might be involved in vascular endothelial dysfunction during sepsis‐induced lung injury.

### 2.3 H3K14 lactylation promoted EC proinflammatory and coagulation activation in response to LPS

**FIGURE 4:**
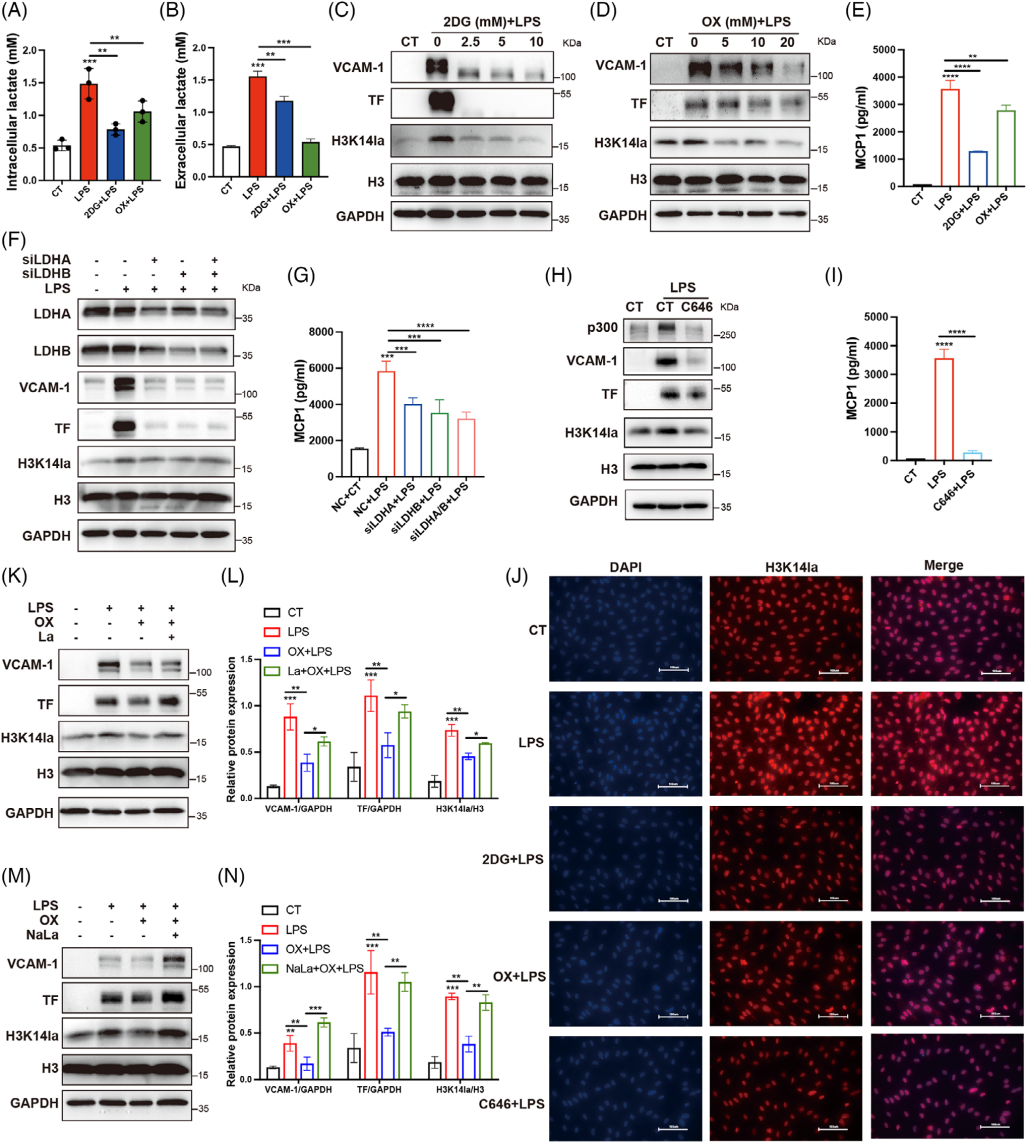
Lactate/H3K14la induced endothelial cell (EC) activation in response to LPS in vitro. (A, B) Intracellular and extracellular lactate levels in HUVECs treated with 2DG or sodium oxamate (OX) before LPS stimulation (*n* = 3). (C, D) Immunoblot analysis of the protein expression levels of VCAM‐1, TF, Kla, and H3K14la in HUVECs treated with 2DG and sodium oxamate (OX) 30 min before LPS stimulation. (E) MCP1 levels in the supernatants were measured by ELISA (*n* = 3). (F, G) Western blot analysis of the protein levels of VCAM‐1, TF, Kla, and H3K14la in response to LPS in HUVECs transfected with or without siLDHA or siLDHB. (G) MCP1 levels in the supernatants were measured by ELISA (*n* = 3). (H) Western blot analysis of the protein levels of VCAM‐1, TF, Kla, and H3K14la in response to LPS in HUVECs with or without C646 pretreatment. (I) MCP1 levels in the supernatants were measured by ELISA (*n* = 3). (J) Representative IF images of H3K14la in HUVECs treated with 2DG, sodium oxamate (OX), or C646 before LPS stimulation. (K‐N) HUVECs were treated with sodium oxamate (OX) before LPS stimulation in the presence of lactate (5 mM) or sodium lactate (5 mM). The protein levels of VCAM‐1, TF, Kla, and H3K14la were analysed by western blotting (*n* = 3).

We further investigated the role of H3K14la in regulating endothelial dysfunction. Upon LPS stimulation, lactate production and the levels of histone Kla and H3K14la were significantly increased (Figure 4A,B; Figure S3A). To confirm the role of histone lactylation in LPS‐induced EC activation, HUVECs were pretreated with 2DG and oxamate prior to LPS stimulation. As depicted in Figure 4A–D, the LPS‐induced increases in both extracellular and intracellular lactate levels, as well as histone Kla and H3K14la levels, were significantly inhibited by 2DG and oxamate (Figure 4A–D,H). Moreover, the upregulation of VCAM‐1 and TF as well as the production of MCP1 were attenuated (Figure 4C–E). LDHA and LDHB play roles in the balance between pyruvate and lactate with different affinities, and the inhibition of both LDHA and LDHB has been shown to completely block lactate production.^8^, ^10^ To verify the effects of lactate production inhibition on lactylation and EC activation, LDHA and LDHB were depleted in HUVECs before LPS stimulation. Depletion of LDHA and LDHB effectively reduced the level of H3K14la and inhibited the upregulation of VCAM‐1, TF, and MCP1 (Figure 4F,G; Figure S3B,C). In addition, C646, an inhibitor of the lysine acetyltransferase p300, which catalyzes the lactylation of lysine,^6^, ^10^ diminished the LPS‐induced increase in H3K14la and upregulated of VCAM‐1, TF, and MCP1 in ECs (Figure 4H–J). To confirm that lactate accumulation plays a role in LPS‐induced EC activation, extracellular lactic acid (La) and sodium lactate (NaLa) were supplemented before the inhibition of intracellular lactate production from glycolysis. The results revealed that the inhibitory effect of oxamate on Kla, H3K14la, VCAM‐1, and TF were at least partially reversed by supplementation with extracellular lactate as well as sodium lactate (Figure 4K–N). In addition, to clarify the role of exogenous lactate in EC activation, we treated HUVECs with lactic acid and sodium lactate. The results revealed that histone Kla and H3K14la levels were increased and that the expression levels of VCAM‐1 and TF as well as the production of MCP1 in HUVECs were increased (Figure S3D–G). These data indicate that the intracellular accumulation of lactate plays a role in EC activation via H3K14la.

The flux of lactate through the cell membrane is mediated by monocarboxylate transporters (MCTs).^21^ To investigate whether exogenous lactate was taken up into ECs via MCTs for histone lactylation, we blocked MCT activity with an MCT inhibitor (AZD3965) and observed that the exogenous lactate‐induced increases in histone Kla and H3K14la in ECs were suppressed by MCT inhibition (Figure S3H–K). These results suggest that extracellular lactate promotes H3K14 lactylation via MCTs.

### 2.4 H3K14la promoted ferroptosis in ECs in response to LPS via transcriptional regulation of TFR and SLC40A1

**FIGURE 5:**
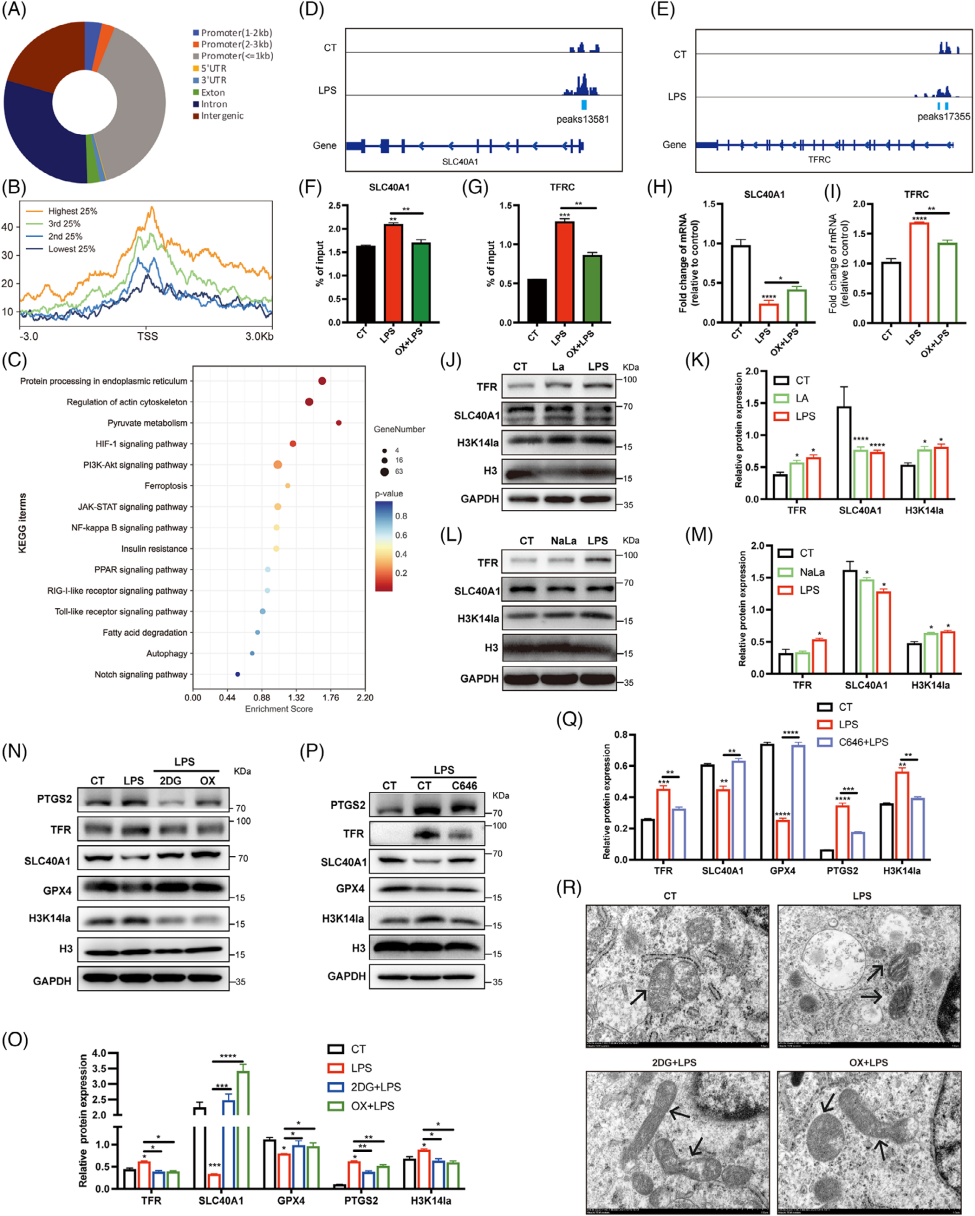
H3K14la promotes ferroptosis in HUVECs by regulating TFR/SLC40A1 expression. (A) Genome‐wide distribution of H3K14la binding peaks in HUVECs. (B) The Cut&Tag peak signals of H3K14la in the four categories of all identified genes based on their mRNA expression levels (the top 25%, the second 25%, the third 25%, and the bottom 25% of RNA‐seq counts). (C) KEGG enrichment of predicted genes based on H3K14la binding peaks. (D, E) Integrative Genomics Viewer (IGV) tracks showing the Cut&Tag signals at the promoter regions of TFRC and SLC40A1. (F, G) ChIP‒qPCR analysis of H3K14la levels at the promoter regions of TFRC and SLC40A1 in HUVECs treated with or without sodium oxamate (OX) before LPS stimulation. (H, I) RT‒qPCR analysis of the mRNA expression levels of TFRC and SLC40A1 in HUVECs pretreated with or without sodium oxamate (OX) before LPS stimulation. (J–M) HUVECs were incubated with LPS or exogeneous lactate (La) and sodium lactate (NaLa). The protein expression levels of TFR, SLC40A1 and H3K14la were analyzed by immunoblotting. (N–Q) HUVECs were pretreated with 2DG, sodium oxamate (OX), or C646 before LPS stimulation. The protein expression levels of PTGS2, TFR, SLC40A1, and GPX4 were analyzed by immunoblotting. (R) Representative transmission electron microscopy (TEM) images showing changes in mitochondrial morphology in HUVECs treated with LPS. The black arrows indicate typical mitochondrial morphology in cells.

To explore the regulatory effects of H3K14la on gene transcription in ECs, we performed high‐throughput Cut&Tag analysis using an H3K14la antibody to identify the transcriptional targets regulated by H3K14la in ECs. A total of 17,363 annotated genome‐binding peaks were identified, 45.62% of which were enriched in promoter regions (Figure 5A). By combining high‐throughput Cut&Tag data with RNA sequencing (RNA‐seq) data from the transcriptome of ECs, all identified genes were divided into four categories based on their expression levels, and the Cut&Tag peak signals of H3K14la on these genes were plotted (Figure 5B). Intriguingly, a positive correlation between H3K14la levels and gene expression at TSSs was observed, strongly suggesting the role of H3K14la in regulating gene transcription in ECs. Kyoto Encyclopedia of Genes and Genomes (KEGG) analysis revealed that the genes regulated by H3K14la were enriched mainly in metabolic pathways, the HIF‐1 signaling pathway, the PI3K‐Akt signaling pathway, and ferroptosis (Figure 5C). Iron overload contributes to bacterial growth in various tissues, and dysregulated iron metabolism leads to ferroptosis, which is a nonapoptotic form of cell death caused by excessive lipid peroxidation.^22^ Integrative genomics viewer (IGV) visual analysis revealed that the enrichment of H3K14la at the promoter regions of the ferroptosis‐related genes TFRC (transferrin receptor) and SLC40A1 (iron‐regulated transporter solute carrier family 40 member 1) upon LPS stimulation was greater than that in the control cells (Figure 5D,E). TFR encoded by TFRC for iron import, and ferroportin (FPN), encoded by SLC40A1 for iron export, are key iron transporters on the cell membrane, and their dysregulation results in increased iron uptake and promotes the accumulation of the labile iron pool, leading to increased lipid peroxidation and thereby ferroptosis.^23^ ChIP‒qPCR revealed that the LPS‐induced increases in H3K14la enrichment at the promoter regions of TFRC and SLC40A1 were reduced by LDHA inhibition (Figure 5F,G). RT‒qPCR revealed that in LPS‐activated ECs, TFR was upregulated and SLC40A1 was downregulated, and these effects were reversed upon LDH inhibition (Figure 5H,I). To confirm the transcriptional regulation of ferroptosis‐related genes by H3K14 lactylation in ECs, extracellular La and NaLa were supplemented, and western blot results revealed that TFR was upregulated and SLC40A1 was downregulated (Figure 5J–M). We further investigated the regulatory role of lactate‐derived H3K14la on key ferroptosis‐related proteins in ECs. Upon the incubation of HUVECs with lactate, sodium lactate, or LPS, the expression of prostaglandin‐endoperoxide synthase 2 (PTGS2), which is related to both ferroptosis and the proinflammatory response of cells, was upregulated (Figure 5N–Q). Moreover, GPX4 uses glutathione to detoxify lipid peroxidation products, essentially protecting cells against ferroptosis.^24^ The downregulation of GPX4 in HUVECs further confirmed the occurrence of lipid peroxidation and ferroptosis upon stimulation with LPS, lactate, and sodium lactate, in accordance with the H3K14la expression data (Figure 5N–Q). As expected, the inhibition of lactate production effectively reduced H3K14la levels (Figure 5N–Q). Moreover, the LPS‐induced downregulation of SLC40A1 and GPX4 and the upregulation of PTGS2 and TFR in HUVECs were reversed upon the inhibition of lactate production (Figure 5N,O). Additionally, the p300 inhibitor C646 attenuated the LPS‐induced changes in ferroptosis‐related proteins (Figure 5P,Q). These results revealed that lactate‐related H3K14 lactylation plays an important role in modulating EC ferroptosis during sepsis. These data revealed the transcriptional regulation of ferroptosis‐related genes by H3K14 lactylation in ECs.

Ferroptosis is characterized by iron‐induced accumulation of lipid reactive oxygen species (ROS) caused by iron‐dependent ROS production, leading to oxidative stress. We observed that LPS stimulation increased the levels of ROS and lipid ROS in ECs in response to LPS stimulation were significantly decreased by the inhibition of lactate production (Figure S4A–E). We further detected ferrous ions in ECs by staining cells with FerroOrange. As shown by the decreased orange fluorescence, excessive ferrous ions in ECs upon LPS stimuli were significantly decreased by 2DG and oxamate (Figure S4A). To confirm the occurrence of ferroptosis in ECs, changes in mitochondrial morphology in LPS‐stimulated HUVECs were examined by transmission electron microscopy (TEM). Mitochondrial atrophy, a decrease in or even the disappearance of the mitochondrial ridge, and an increase in the density of the mitochondrial membrane were observed, indicating lipid peroxidation in the mitochondria (Figure 5R). In addition, we analyzed the effect of lactate on pyroptosis and found that the levels of total gasdermin D and the N‐terminus of gasdermin D in HUVECs in HUVEC were not affected upon lactate stimulation, indicating that lactate has no effect on pyroptosis (Figure S4F). These results revealed that lactate‐related H3K14 lactylation plays an important role in modulating EC ferroptosis during sepsis.

### 2.5 H3K14la‐mediated ferroptosis promoted EC activation in sepsis‐related lung injury

**FIGURE 6:**
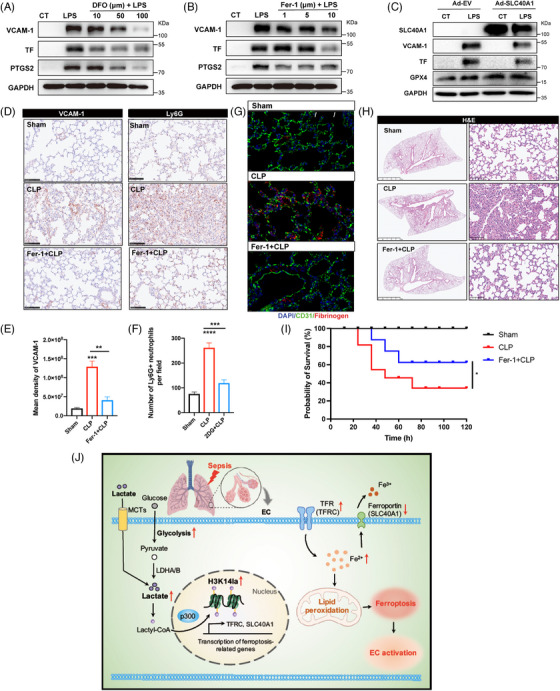
Ferroptotic stress promotes endothelial dysfunction in sepsis‐induced lung injury. (A, B) HUVECs were treated with DFO and ferrostatin‐1 before LPS stimulation. The expression levels of VCAM‐1, TF, and PTGS2 were determined by western blotting. (C) SLC40A1‐overexpressing HUVECs were stimulated with LPS for 4 h. The expression levels of SLC40A1, VCAM‐1, TF, and GPX4 were analyzed by western blotting. Adenovirus carrying empty vehicle (Ad‐EV) was used as a vehicle control. (D–F) Mice were i.p. injected with ferrostatin‐1 (Fer‐1) prior to the CLP‐induced sepsis model. The expression of VCAM‐1 and the infiltration level of Ly6G+ neutrophils in mouse lung tissues were analyzed by immunohistochemical (IHC) staining and quantified by pathologists in a blinded manner. (G) Immunofluorescence staining of fibrinogen in the lung tissues of different groups. Fibrinogen is stained red; the pulmonary EC marker CD31 is stained green; and the nuclei are stained blue. (H) Lung tissue injury was evaluated by H&E staining. (I) Survival rates were compared among the sham, cecal ligation and puncture (CLP), and CLP + Fer‐1 groups using the Kaplan–Meier test. (J) Graphic summary of the study findings (*n* = 15 for each group).

Ferroptosis has been reported to initiate dysregulated immune responses in inflammatory diseases.^25^ To investigate the role of ferroptosis in LPS‐induced EC activation, two representative ferroptosis inhibitors, ferrostatin‐1 (Fer‐1) and deferoxamine (DFO), were used; the former is a synthetic antioxidant, and the latter is an effective iron chelator. Compared with LPS pretreatment, Fer‐1 and DFO pretreatment improved cell viability (Figure S5A,B). In addition, the LPS‐induced upregulations of VCAM‐1, TF and PTGS2 were significantly inhibited by both Fer‐1 and DFO (Figure 6A,B). Among others, the SLC40A1‐encoded protein FPN is a transmembrane iron transporter that plays a critical role in maintaining iron homeostasis.^26^ Excess iron leads to the degradation of FPN, limiting cellular iron efflux.^27^ To further explore the role of SLC40A1 in ECs, we overexpressed SLC40A1 in HUVECs before LPS stimulation. The results revealed that SLC40A1 overexpression led to the upregulation of GPX4 expression in HUVECs stimulated with LPS (Figure 6C), indicating the positive role of SLC40A1 in ferroptosis resistance. Moreover, LPS‐induced upregulation of VCAM‐1 and TF was downregulated in HUVECs overexpressing SLC40A1, revealing its inhibitory effects on LPS‐induced EC activation (Figure 6C). These data suggest a role for SLC40A1‐mediated ferroptosis in promoting EC activation in response to LPS.

To confirm the importance of ferroptosis in vascular dysfunction during sepsis‐induced lung injury, mice were treated with Fer‐1 before LPS challenge. The results revealed that the increase in VCAM‐1 expression and neutrophil infiltration in the pulmonary microvascular bed was strongly mitigated upon ferroptosis inhibition (Figure 6D–F; Figure S5C–E). Additionally, the levels of fibrinogen in the lung tissues were limited by Fer‐1 treatment (Figure 6G, Figure S5F,G). Lung tissue injury in septic mice was also obviously alleviated in Fer‐1‐treated mice (Figure 6H, Figure S5H). Moreover, the administration of Fer‐1 significantly improved the survival rate of septic mice (Figure 6I). These results revealed the important role of ferroptosis in sepsis‐related vascular endothelial activation and lung injury.

## 3 DISCUSSION

In the present study, we identified protein lactylation as a novel regulatory role of lactate in EC activation during sepsis‐induced lung injury. As an important clinical indicator, serum lactate is strongly correlated with the progression, severity, and mortality of sepsis, although the regulatory role of lactate during sepsis remains unclear. Recent studies have shown that the lactate content in cells can induce post‐translational lactylation of lysine residues in both histone and non‐histone proteins. Our study, for the first time, discovered the regulatory effects of glycolysis‐derived H3K14la on ferroptosis in sepsis‐induced lung injury and revealed that H3K14la activated ECs by modulating the gene transcription of TFRC and SLC40A1. The inhibition of lactate production decreased H3K14la levels, which suppressed EC ferroptosis and thereby reduced EC activation, alleviating sepsis‐induced lung injury (Figure 6J). Our study provides a new illustration of the role of the glycolysis/H3K14la/ferroptosis axis in regulating EC activation and lung injury during sepsis‐induced ARDS.

Protein lactylation is a novel PTM that has been identified to play regulatory roles in different disease conditions. Immune disorders play essential roles in sepsis‐induced lung injury.^28^ Moreover, metabolic disorders characterized by overwhelmed glycolysis and impaired lipid metabolism during sepsis have detrimental effects on organ functions.^29^, ^30^ Inhibition of glycolysis effectively alleviates polymicrobial sepsis‐induced cardiomyopathy and improves septic survival by regulating the inflammatory response and apoptotic signaling.^31^ Our present study revealed that lactylated proteins are enriched in energy metabolism processes, including the TCA cycle, FAO, lipid metabolic processes, and glycolic processes. Our findings provide a new epigenetic‒metabolic link in which lactylation derived from glycolysis may regulate metabolic activity and serve as a sensor for metabolic feedback. Exploring protein lactylation in sepsis‐related etiology and pathophysiology holds great promise, thereby contributing to a better understanding of the roles of lactate in sepsis‐induced organ dysfunction.

As an epigenetic modification, histone lactylation widely regulates chromatin‐based gene transcription. To date, several studies have reported the regulatory roles of histone lactylation in disease progression. H3K18la promotes tumorigenesis by facilitating the YTHDF2‐mediated recognition of N6‐methyladenosine (m6A) and the degradation of the tumor suppressor genes PER1 and TP53 in ocular melanoma.^10^ In addition, the roles of histone lactylation in regulating macrophage functions have been reported in several different disease models. H3K18la drives the M2 polarization of macrophages by activating the transcription of M2‐related genes.^8^ B‐cell adapter for PI3K (BCAP), an adapter protein of TLR signaling, promotes the transition of inflammatory macrophages to reparative macrophages by inducing histone lactylation.^9^ Moreover, in the context of lung fibrosis, lactate released from lung myofibroblasts promotes the transcription of profibrotic genes in macrophages via histone lactylation.^32^ In our study, we demonstrated that H3K14la regulates iron metabolism‐related genes to drive EC activation during sepsis‐related ARDS, revealing a novel mechanism by which glycolysis‐derived lactate in regulates sepsis‐related EC dysfunction.

Ferroptosis, an iron‐dependent form of cell death, is a pathophysiological process closely related to dysregulated metabolism, such as lipid metabolism and TCA cycle disturbance. Excessive accumulation of intracellular free iron interacts with hydrogen peroxide via the Fenton reaction, leading to lipid peroxidation of poly‐unsaturated fatty acids and initiating ferroptosis.^23^ Iron overload initiates ferroptosis and is known to be an important factor for maintaining bacterial growth in various tissues.^33^, ^34^, ^35^ The iron chelator deferoxamine plays a protective role in early sepsis.^36^, ^37^ Recent studies have reported the involvement of ferroptosis in EC dysfunction associated with diverse pathophysiological processes and diseases, such as atherosclerosis,^38^ blood‐brain barrier injury,^39^ and diabetes.^40^ Moreover, the inhibition of ferroptosis can alleviate sepsis‐induced vascular leakage, suggesting the regulatory role of ferroptosis in EC functions under septic conditions.^41^ Our study revealed that ferroptosis inhibition attenuated LPS‐induced proinflammatory activation in ECs. In addition, we observed the downregulation of GPX4 in response to LPS stimulation both in vivo and in vitro. GPX4‐mediated lipid peroxidation, an auto‐oxidative process induced by stress, contributes to the progression of various types of regulated cell death.^42^ It has been reported that GPX4 decreases lipid peroxidation and negatively regulates both ferroptosis and pyroptosis in lethal polymicrobial sepsis.^43^, ^44^ Ferroptotic cell rupture is mediated by plasma membrane pores in a process that is similar to cell lysis in pyroptosis and necroptosis.^45^ The associations between different types of programmed cell death still need further exploration.

Moreover, we investigated the impact of histone lactylation on regulating the transcription of ferroptosis‐related genes, revealing the relationship between lactate and ferroptosis. Consistently, the accumulation of intracellular ferrous iron and lipid peroxides in cystic fibrosis airway epithelial cells is positively correlated with the level of lactate dehydrogenase, the key enzyme that converts pyruvate to lactate.^46^ Interestingly, in contrast to previous studies demonstrating gene transcriptional activation by histone lactylation, we found that LPS‐induced H3K14la in ECs activates TFRC transcription but inhibits SLC40A1 transcription. Notably, changes in the PTMs of histones may affect both the transcriptional activation and inhibition of candidate genes. Histone modification influences the interactions between chromatin and DNA‐binding proteins, such as transcription factors, in a manner dependent on the modification locus.^47^ Therefore, the potential transcription factors responsible for H3K14la‐related regulation of ferroptosis‐related genes need to be further explored.

In this study, we revealed that sepsis‐induced lactate production induced EC proinflammatory and coagulation activation‐associated lung injury via H3K14la‐mediated transcription of the ferroptosis‐related genes TFR and SLC40A1, indicating the role of lactate in regulating EC‐related pathogenesis of lung injury and providing a new therapeutic strategy for the treatment of sepsis‐associated ARDS. However, our present study has several limitations that should be noted. First, in addition to lactylation, H3K14 undergoes other epigenetic modifications, such as acetylation, methylation, and crotonylation, which affect the expression of target genes. Site mutations should be generated for H3K14 to avoid the interference of histone lactylation from other modifications in future studies. Second, the crosstalk between different modifications also needs to be explored. Third, investigations of histone deacetylases 1–3 (HDAC1–3), which are identified as deacetylases, will help reveal the role of H3K14la in sepsis‐induced EC activation. Fourthly, given the presence of both intracellular and extracellular lactate in the in vivo microenvironment, it is essential to isolate vascular ECs from the lung tissues of both control and septic mice. This will allow for a more definitive validation of our findings concerning the regulatory role of glycolysis/H3K14la/ferroptosis in sepsis. Ultimately, direct intervention targeting H3K14la to evaluate its impact on sepsis associated lung injury via endothelial cells is urgent while technically challenging, and there is ample scope for enhancing our understanding of its underlying mechanisms.

## 4 METHODS

### 4.1 Patient enrolment

Patients diagnosed with sepsis in the emergency department of Shanghai Ruijin Hospital between October 31, 2021, and May 20, 2022, were included in this study. The study was approved by the Ethics Committee of Ruijin Hospital (No. 20210101). All investigations followed the institution's guidelines for human studies and adhered to the ethical principles of the Declaration of Helsinki. Informed consent was obtained from all participants. The inclusion criteria were: (1) age between 18 and 90 years, (2) diagnosis based on the Sepsis 3.0 criteria, and (3) hospital stay exceeding 24 h. The exclusion criteria included: (1) discharge or death within 24 h of admission, (2) participation in other clinical studies, (3) emergency surgery post‐admission, (4) malignant tumors, (5) pregnancy or lactation, and (6) missing critical clinical data. A total of 20 healthy volunteers and 38 patients with sepsis were ultimately enrolled. The baseline characteristics of the healthy volunteers and sepsis patients have been reported in previous research.^48^

### 4.2 Animals

Male C57BL/6J mice (6–10 weeks, 20–30 g) were randomly assigned to experimental and control groups. The mice were maintained on a regular chow diet in a controlled environment with specific temperature and light‐dark cycles. All animal protocols were approved by the Animal Ethics Committee of Ruijin Hospital Affiliated with Shanghai Jiaotong University School of Medicine (No. 092) and adhered to the International Guidelines for Care and Use of Laboratory Animals (National Academy of Sciences Health Publication No. 85–23, revised in 1996).

Mice were initially anesthetized via intraperitoneal injection of 1% phenobarbital (1 mg/kg). Sepsis was induced through a cecal ligation and puncture (CLP) procedure. After anesthesia, abdominal fur was shaved, and the area was sterilized with 70% ethanol. A 0.5 cm midline incision was made to expose the cecum, which was ligated below the ileocecal valve. Two punctures were made at the top and bottom of the cecum using an 18‐G needle, and a small amount of fecal material was extruded. The cecum was repositioned in the abdominal cavity, and 50 mL/kg of physiological saline (0.9%) was administered subcutaneously. Mice were allowed to recover and were sacrificed at designated time points.

To induce endotoxemia and related organ damage, mice were administered intraperitoneal (i.p.) injections of LPS (5 mg/kg body weight). Vehicle control mice received equal volumes of 0.9% saline via i.p. injection. In the intervention groups, sodium oxamate (500 mg/kg body weight) or the glycolysis inhibitor 2DG (250 mg/kg body weight) was administered i.p. 1 h prior to sepsis induction to inhibit lactate production. Mice were sacrificed, and blood samples were collected at designated time points. Tissues were either snap‐frozen and stored at −80°C or fixed in 4% formalin for further analysis.

### 4.3 Cell culture

Human umbilical vein endothelial cells (HUVECs) were isolated from human umbilical cords as described in previous studies.^18^ HUVECs were cultured in EGM‐2MV medium (CC‐3202, Lonza) supplemented with 10% fetal bovine serum (FBS, CC‐4102B, Lonza) and antibiotics (streptomycin and penicillin). The cells were maintained in a humidified incubator at 37°C with 5% CO_2_. HUVECs from passages 1 to 4 were used for all experiments. The use of HUVECs followed human subject guidelines set by Ruijin Hospital, Shanghai Jiao Tong University.

### 4.4 LC‒MS/MS Analysis

Liquid chromatography‐tandem mass spectrometry (LC–MS/MS) was conducted at PTM Biolabs. Tryptic peptides were dissolved in solvent A (0.1% formic acid, 2% acetonitrile in water) and loaded onto a custom‐made reverse‐phase analytical column (25 cm long, 100 µm i.d.). The peptides were separated using a gradient of 7% to 24% solvent B (0.1% formic acid in acetonitrile) over 42 min, followed by 24% to 32% in 12 min, increased to 80% in 3 min, and held at 80% for the last 3 min. The flow rate was kept at 450 nL/min using a nanoElute UHPLC system (Bruker Daltonics).

Peptides were ionized and analyzed on a timsTOF Pro (Bruker Daltonics) mass spectrometer using electrospray ionization with a 1.75 kV voltage. Precursor and fragment ions were detected on the TOF detector, with MS/MS scans ranging from 100 to 1700 m/z. The instrument operated in parallel accumulation serial fragmentation (PASEF) mode, and 10 PASEF‐MS/MS scans were performed per cycle. Dynamic exclusion was set at 30 seconds.

### 4.5 Database search

MS/MS data were analyzed using MaxQuant (v.1.6.15.0). Spectra were matched to the Mus_musculus_10090_SP_20201214.fasta database (17,063 entries) concatenated with a reverse decoy database. Trypsin/P was set as the cleavage enzyme, allowing up to four missed cleavages. Mass tolerance for precursor ions was 20 ppm in both the first and main searches, and fragment ion tolerance was set at 0.02 Da. Carbamidomethylation of Cys was a fixed modification, while protein N‐terminal acetylation, Met oxidation, and Lys lactylation were variable modifications. The false discovery rate (FDR) was set at less than 1%.

### 4.6 CUT&Tag assay

The CUT&Tag assay was performed by Jiayin Biotechnology Ltd. HUVECs were bound to Concanavalin A‐coated magnetic beads (Bangs Laboratories) for 10 min at room temperature. Bead‐bound cells were incubated with a primary antibody against H3K14la (1:50 dilution, Anti‐Lactyl‐Histone H3 (Lys14), PTM‐1414) or a normal rabbit IgG control (Millipore cat. No. 12–370). After incubation with a secondary antibody (anti‐rabbit IgG, Millipore AP132), cells were incubated with the pA‐Tn5 adapter complex for transposon activation and tagmentation. DNA was purified and amplified for library construction. Libraries were cleaned up using XP beads (Beckman Counter) and analyzed with the Agilent 4200 TapeStation for size distribution. Sequencing was conducted on an Illumina NovaSeq 6000 using 150 bp paired‐end sequencing.

### 4.7 Chromatin immunoprecipitation–quantitative PCR (ChIP‒qPCR)

Cells were crosslinked with 1% formaldehyde in 9 mL of culture medium at room temperature for 10 min. Crosslinking was stopped by adding glycine to a final concentration of 0.125 M and incubating for 5 min. Chromatin extraction was carried out using the SimpleChIP Enzymatic Chromatin IP Kit (#9003) following the manufacturer's protocol. After sonication, the chromatin was immunoprecipitated with an anti‐Lactyl‐Histone H3 (Lys14) (PTM1414) antibody overnight at 4°C. Following cross‐link reversal and DNA purification, the eluted DNA was used for qPCR with specific target region primers (Table S1). Quantification was done using the comparative CT method and normalized to 2% input.

### 4.8 Immunofluorescence (IF) staining

For tissue IF staining, deparaffinized lung sections were heated at 98°C for 10 min for antigen retrieval and blocked with 10% goat serum at room temperature for 1 h. Sections were incubated with the relevant antibodies (as detailed in the Methods section) overnight at 4°C. After incubation with Alexa Fluor‐conjugated secondary antibodies (Servicebio) at room temperature for 1 h, the nuclei were stained with DAPI (Servicebio) for 5 min. Samples were mounted with mounting medium (Sigma) and stained with diaminobenzidine for microscopic examination (DS‐U3, Nikon).

For HUVECs, cells were fixed in 4% paraformaldehyde for 15 min, permeabilized with 0.1% Triton X‐100 for 20 min, and blocked with 3% BSA for 30 min. Cells were incubated with primary antibodies overnight at 4°C, followed by incubation with fluorescein‐labeled secondary antibodies for 1 h at room temperature. Finally, the cells were mounted with Antifade Mounting Medium with DAPI, and images were captured using a Nikon DS‐U3 microscope.

### 4.9 Western blot analysis

Total protein from lung tissue or cells was extracted using RIPA lysis buffer with protease and phosphatase inhibitors. Proteins (20 µg) were separated by 12.5% SDS‐PAGE and transferred to PVDF membranes (Millipore). After blocking in 5% BSA for 1 h at room temperature, membranes were incubated with primary antibodies (as described in the Methods section) overnight at 4°C. Membranes were washed and incubated with secondary antibodies (1:10,000) for 1 h at 37°C. After further washes, signals were detected using an ECL substrate (Tanon), and protein bands were visualized using a Bio‐Rad imaging system.

### 4.10 Enzyme‐Linked Immunosorbent Assay (ELISA)

Serum IL‐6, sE‐selectin, sVCAM‐1, sICAM‐1 (human), IL‐6, TNF‐α (mouse), and MCP‐1 (HUVEC supernatants) were measured using ELISA kits (MultiSciences Biotechnology) according to the manufacturer's instructions.

### 4.11 Immunohistochemical (IHC) staining

For IHC staining, fixed lung tissues were dehydrated, paraffin‐embedded, and sectioned (5 µm). Sections were treated with endogenous peroxidase and blocked with non‐specific proteins before incubation with primary antibodies overnight at 4°C. PBS was used as the negative control. After incubation with a biotinylated secondary antibody for 1 h at room temperature, sections were stained with diaminobenzidine for microscopic examination (Nikon DS‐U3).

### 4.12 Measurement of lactate and LDH levels

Lactate levels in human serum, mouse plasma, cell supernatants, and homogenates were measured using the L‐Lactate Assay Kit (Eton Bioscience) per the manufacturer's protocol, with absorbance measured at 490 nm. Serum LDH concentrations were determined using commercial kits (Nanjing Jiancheng Bioengineering Institute).

### 4.13 Small interfering RNA transfection and adenoviral transduction

HUVECs were transfected with siRNA (GenePharma) or negative control siRNA using HiFect (QIAGEN) as per the manufacturer's instructions. siRNA sequences are listed in Table S1. For SLC40A1 overexpression, an adenovirus carrying the SLC40A1 gene (pcADV‐EF1‐mNeonGreen‐CMV vector, OBiO) was used. HUVECs were transfected at 80%–90% confluence, with adenovirus incubation for 6 h, followed by a 42‐h incubation in fresh medium. Transfection efficiency (> 90%) was confirmed before experiments.

### 4.14 Transmission electron microscopy (TEM)

After experiments, HUVECs were prefixed in 2.5% glutaraldehyde phosphate buffer (0.1 M, pH 7.4) at 4°C overnight, postfixed in 2% osmium tetroxide, and embedded in Epon812 (Merck) following dehydration. Ultrathin sections (60 nm) were stained with uranyl acetate and lead citrate and imaged using a TEM (FEI).

### 4.15 ROS, lipid ROS, and FerroOrange staining

Intracellular ROS levels were measured using the reactive oxygen species assay kit (DOJINDO). At the conclusion of the experiments, the medium was removed, and 5 µM dichlorodihydrofluorescein diacetate was added to each well, followed by incubation for 30 min at 37°C. After washing the cells with PBS, ROS fluorescence was visualized using a fluorescence microscope or quantified by flow cytometry.

For intracellular ferrous ions and lipid ROS detection in HUVECs, the cells were suspended in fresh Hank's balanced salt solution (HBSS) and stained with 5 µM C11‐BODIPY for 60 min or 10 µM FerroOrange for 30 min, both at 37°C. After washing, fluorescence imaging for lipid ROS and ferrous ions was performed using a fluorescence microscope or quantified by flow cytometry.

### 4.16 Statistics

Data are presented as means ± SD unless otherwise stated. Results were based on at least three independent experiments. Unpaired two‐tailed *t*‐tests were used to compare two groups, and one‐way ANOVA followed by Bonferroni correction was used for multiple groups. Analyses were performed using GraphPad Prism 9 and FlowJo software. *p* ≤ 0.05 was considered statistically significant.

## AUTHOR CONTRIBUTIONS

Erzhen Chen, Ranran Li, and Ying Chen conceived and directed the project. Fangchen Gong and Wen Xu designed and performed most of the experiments, analyzed the data, and wrote the manuscript. Xiangtao Zheng and Rongli Xie performed the bioinformatic analysis of LC‐MS/MS assay and CUT&Tag assay. Wenbin Liu and Lei Pei isolated primary endothelial cells from fresh human umbilical cords. Ming Zhong, Wen Shi, Hongping Qu, Enqiang Mao, and Zhitao Yang helped with data statistical analysis. Fangchen Gong, Wen Xu, and Xiangtao Zheng shared the first author position. All authors discussed and commented on the manuscript and agreed for the submission of the manuscript.

## CONFLICT OF INTEREST STATEMENT

The authors declare no conflicts of interest.

## ETHICS STATEMENT AND CONSENT TO PARTICIPATE

All human sample study protocols were approved by the Ethics Committee of Ruijin Hospital (no. 20210101).

## Supporting information



Supporting Information

## Data Availability

The datasets used and/or analyzed during the current study are available from the corresponding author on reasonable request. The mass spectrometry proteomics data have been deposited to the ProteomeXchange Consortium (http://proteomecentral.proteomexchange.org) via the iProX partner repository with the dataset identifier PXD056805.
