# Supplementary material for: H3K14la drives endothelial dysfunction in sepsis‐induced ARDS by promoting SLC40A1/transferrin‐mediated ferroptosis
Source: MedComm (2020). 2025 Jan 14;6(2):e70049. doi: 10.1002/mco2.70049 (PMC11733091; doi:10.1002/mco2.70049)
Supplement: Supplementary file 1 — Supporting Information [file MCO2-6-e70049-s001.docx]

**H3K14la drives endothelial dysfunction in sepsis-induced ARDS via promoting SLC40A1/TFR-mediated ferroptosis**

**Running title: H3K14la facilitates EC dysfunction via ferroptosis**

Fangchen Gong^1^*, Xiangtao Zheng^1^*, Wen Xu^2^*, Rongli Xie^3^*, Wenbin Liu^1^, Lei Pei^1^, Ming Zhong^2^, Wen Shi^1^, Hongping Qu^2^, Enqiang Mao^1^, Zhitao Yang^1^, Ranran Li^2#^, Erzhen Chen ^1#^, Ying Chen ^1#^

1 Department of Emergency, Ruijin Hospital, Shanghai Jiao Tong University School of Medicine, Shanghai, P.R. China.

2 Department of Critical Care Medicine, Ruijin Hospital, Shanghai Jiao Tong University School of Medicine, Shanghai, P.R. China.

3 Department of General Surgery, Ruijin Hospital Lu Wan Branch, Shanghai Jiaotong University School of Medicine, Shanghai, China

* These authors contributed equally to this work.

# Correspondence to: bichatlion@163.com (Y.C.), cez10732@rjh.com.cn (E.C.), ranranli@shsmu.edu.cn (R.L.),

**Supplemental materials**

**Table S1. Lactylated non-histone proteins in lung tissues that were significantly different between septic and control mice**

| **Protein accession** | **Protein description** | **Gene name** | **P value** |
| --- | --- | --- | --- |
| P97315 | Cysteine and glycine-rich protein 1 | Csrp1 | 0.00049236 |
| Q9D8B3 | Charged multivesicular body protein 4b | Chmp4b | 0.00068576 |
| P61979 | Heterogeneous nuclear ribonucleoprotein K | Hnrnpk | 0.00084838 |
| Q60749 | KH domain-containing, RNA-binding, signal transduction-associated protein 1 | Khdrbs1 | 0.00109264 |
| P10107 | Annexin A1 | Anxa1 | 0.00180338 |
| O08553 | Dihydropyrimidinase-related protein 2 | Dpysl2 | 0.0029391 |
| P20152 | Vimentin | Vim | 0.00325251 |
| P26443 | Glutamate dehydrogenase 1 | Glud1 | 0.00405518 |
| Q570Y9 | DEP domain-containing mT | Deptor | 0.00516239 |
| P63158 | High mobility group protein B1 | Hmgb1 | 0.00685169 |
| P07724 | Albumin | Alb | 0.00774362 |
| Q8R081 | Heterogeneous nuclear ribonucleoprotein L | Hnrnpl | 0.00801601 |
| P48678 | Prelamin-A/C | Lmna | 0.00823851 |
| Q6URW6 | Myosin-14 | Myh14 | 0.00863802 |
| P09405 | Nucleolin | Ncl | 0.00964019 |
| O70400 | PDZ and LIM domain protein 1 | Pdlim1 | 0.01088997 |
| P63038 | 60 kDa heat shock protein | Hspd1 | 0.01164169 |
| Q3U0V1 | Far upstream element-binding protein 2 | Khsrp | 0.01399147 |
| P23198 | Chromobox protein homolog 3 | Cbx3 | 0.01538285 |
| P14824 | Annexin A6 | Anxa6 | 0.01632117 |
| Q61029 | Lamina-associated polypeptide 2 | Tmpo | 0.01818678 |
| Q03265 | ATP synthase subunit alpha | Atp5f1a | 0.01849513 |
| Q60710 | Deoxynucleoside triphosphate triphosphohydrolase | Samhd1 | 0.0187284 |
| Q9D0K2 | Succinyl-CoA:3-ketoacid coenzyme A transferase 1 | Oxct1 | 0.01909644 |
| P08249 | Malate dehydrogenase | Mdh2 | 0.03382619 |
| Q8K2B3 | Succinate dehydrogenase | Sdha | 0.03415468 |
| Q9CQL1 | Protein mago nashi homolog 2 | Magohb | 0.03729491 |
| Q63918 | Caveolae-associated protein 2 | Cavin2 | 0.03745938 |
| Q6IRU2 | Tropomyosin alpha-4 chain | Tpm4 | 0.03858973 |
| Q61792 | LIM and SH3 domain protein 1 | Lasp1 | 0.03923728 |
| P01942 | Hemoglobin subunit alpha | Hba | 0.04593148 |
| O54724 | Caveolae-associated protein 1 | Cavin1 | 0.04811908 |
| Q9DCT8 | Cysteine-rich protein 2 | Crip2 | 0.04855192 |
| P50543 | Protein S100-A11 | S100a11 | 0.05015484 |
| Q64727 | Vinculin | Vcl | 0.05189038 |
| Q7TPR4 | Alpha-actinin-1 | Actn1 | 0.05534955 |
| Q8QZT1 | Acetyl-CoA acetyltransferase | Acat1 | 0.05681241 |
| P58771 | Tropomyosin alpha-1 chain | Tpm1 | 0.05816741 |
| P48962 | ADP/ATP translocase 1 | Slc25a4 | 0.05878337 |
| Q9R0P5 | Destrin | Dstn | 0.05901876 |
| P60710 | Actin, cytoplasmic 1 | Actb | 0.0593597 |
| Q8BFZ3 | Beta-actin-like protein 2 | Actbl2 | 0.0593597 |
| Q05920 | Pyruvate carboxylase | Pc | 0.05967527 |
| Q8BFR5 | Elongation factor Tu | Tufm | 0.05987824 |
| Q8VEK3 | Heterogeneous nuclear ribonucleoprotein U | Hnrnpu | 0.06051508 |
| P16546 | Spectrin alpha chain, non-erythrocytic 1 | Sptan1 | 0.06272197 |
| P51881 | ADP/ATP translocase 2 | Slc25a5 | 0.06904269 |
| P68369 | Tubulin alpha-1A chain | Tuba1a | 0.07276634 |
| P08074 | Carbonyl reductase [NADPH] 2 | Cbr2 | 0.07411593 |
| P56382 | ATP synthase subunit epsilon | Atp5f1e | 0.07564584 |
| P49817 | Caveolin-1 | Cav1 | 0.07652705 |
| Q9CZU6 | Citrate synthase | Cs | 0.0779236 |
| Q00915 | Retinol-binding protein 1 | Rbp1 | 0.07976497 |
| P05202 | Aspartate aminotransferase | Got2 | 0.08063298 |
| Q9DB20 | ATP synthase subunit | Atp5po | 0.08250674 |
| Q62261 | Spectrin beta chain, non-erythrocytic 1 | Sptbn1 | 0.09368013 |
| P16858 | Glyceraldehyde-3-phosphate dehydrogenase | Gapdh | 0.09557488 |
| P22599 | Alpha-1-antitrypsin 1-2 | Serpina1b | 0.096898 |

**Table S2 Targeting sequences for siRNA, primers for ChIP-qPCR, and primers for qPCR**

|  | sense | antisense |
| --- | --- | --- |
| siRNA targeting sequences |  |  |
| LDHA-Homo-234 | CUGGCAAAGACUAUAAUGUTT | ACAUUAUAGUCUUUGCCAGTT |
| LDHA-Homo-812 | CCAGUUUCCACCAUGAUUATT | UAAUCAUGGUGGAAACUGGTT |
| LDHA-Homo-104 | GCCAUCAGUAUCUUAAUGATT | UCAUUAAGAUACUGAUGGCTT |
| LDHB-Homo-314 | GGGAGCUUAUUUCUUCAGATT | UCUGAAGAAAUAAGCUCCCTT |
| LDHB-Homo-583 | CGUGAUUGGAAGUGGAUGUTT | ACAUCCACUUCCAAUCACGTT |
| LDHB-Homo-876 | GUGUGGCUGAUCUUAUUGATT | UCAAUAAGAUCAGCCACACTT |
|  |  |  |
| Primers for ChIP-qPCR |  |  |
| TFRC-a site | AGCTGCAGAATCCAGTCCCC | GCTGTGCTGGATTGATGGGC |
| TFRC-b site | CTCCCTATGGCTGCTGGACA | ATAAACCGCCGGTTAGGGGC |
| TFRC-c site | AGCATATAAGCATGGGGTAGCCA | AGGTTGGGAGGGTGGGTAAG |
| TFRC-d site | TGGAAAAAGCTGCAGAATCCAGT | AGAGGGCTGTGCTGGATTGA |
| TFRC-e site | AGCATGGGGTAGCCAAATACAA | TTAAGGTTGGGAGGGTGGGT |
| SLC40A1-a site | ACGAGCTCCCGTCAACCTT | AAAACCCGGGGAGTGGAAC |
| SLC40A1-b site | AGCTTGGTGGGTGGGGATTC | TGCAGTCCGGGGAAGGAAAG |
| SLC40A1-c site | ATCAAAGGGCAGGAAGGGGG | TCCAGGACGGATTTGGAGGC |
| SLC40A1-d site | CTTCCCCGGACTGCATAGCA | GCTGCGGGACTTCACCTTTG |
|  |  |  |
| Primers for qPCR |  |  |
| GAPDH | GGTGAAGGTCG GAGTCAACG | CAAAGTTGTCATGGATGGACC |
| VCAM1 | ACCACATCTACGCTGACAATGAATCC | AACACTTGACTGTGATCGGCTTCC |
| MCP1 | CCCCAGTCACCTGCTGTTAT | CAGATCTCCTTGGCCACAAT |
| TF | ACGAGATTGTGAAGGATGTGAAGCAG | TGTCTCCAGGTAAGGTGTGAACTCTG |
| TFR | CGCTGGTCAGTTCGTGATT | ATGAAAGCAGTTGGCTGTTGTA |
| SLC40A1 | CCTGGAGGGAACTCATCT AAT | CACAAG TAGGCTCTTGCTCAT |

**Table 3. The detail of reagents and resources.**

| REAGENT or RESOURCE | SOURCE | IDENTIFIER |
| --- | --- | --- |
| **Antibodies** |  |  |
| Rabbit anti-Lactyl-Histone H3 (Lys14) | PTM Biolabs | Cat# PTM-1414 |
| Rabbit anti-L-Lactyl Lysine | PTM Biolabs | Cat# PTM-1401 |
| Rabbit anti-VCAM-1 (EPR5047) | Abcam | Cat# ab134047; RRID: AB_2721053 |
| Rabbit anti-Fibrinogen beta chain | Abcam | Cat# ab92510; RRID: AB_10562821 |
| Rat anti-Ly6G | Abcam | Cat#; ab238132 |
| Rabbit anti-Transferrin Receptor | Abcam | Cat# ab214039; RRID: AB_2904534 |
| CD31 | Abcam | Cat# ab76533, RRID: AB_1523298 |
| Alexa Fluor®647 donkey anti-rabbit lgG(H+L) | Abcam | Cat# ab150075 |
| Rabbit anti-Histone H3 | Cell Signaling Technology | Cat# 4499; RRID: AB_10544537 |
| Rabbit anti-PTGS2 | Cell Signaling Technology | Cat# 12282; RRID: AB_2571729 |
| Mouse anti-TF (H-9) | Santa Cruz | Cat# sc-374441; RRID: AB_11008609 |
| Mouse anti-GAPDH | Proteintech | Cat# 60004-1-Ig; RRID: AB_2107436 |
| Rabbit anti-SLC40A1 | Novus | Cat# NBP1-21502; RRID: AB_1660490 |
| Rabbit anti-GPX4 | Signalway Antibody | Cat# 32506 |
| DAPI | Beyotime Biotechnology | Cat# C1002 |
| Alexa Fluor 555 Donkey anti Rabbit 1 1/500 | Thermo Fisher | Cat# A-31572; RRID: AB_162543 |
| **Chemicals, Peptides, and Recombinant Proteins** |  |  |
| Sodium oxamate | Selleck | Cat#S6871 |
| 2-Deoxy-D-glucose | Selleck | Cat#S4701 |
| Ferrostatin-1 | Selleck | Cat#S7243 |
| Deferoxamine mesylate | Selleck | Cat#S5742 |
| C646 | Selleck | Cat#S7152 |
| AZD3965 | Selleck | Cat#S7339 |
| LPS (O111:B4) | Sigma-Aldrich | Cat#L2630 |
| **Critical Commercial Assays** |  |  |
| L- Lactate Assay kit | Eton Bioscience | Cat#1200011002 |
| Mouse IL-6 ELISA Kit | Multi Science | Cat#70-EK206/3-96 |
| Mouse TNF⍺ ELISA Kit | Multi Science | Cat#70-EK282/4-96 |
| Ferrorange | DOJINDO | Cat#F374 |
| ROS Assay Kit -Highly Sensitive DCFH-DA | DOJINDO | Cat#R252 |
| Liperfluo | DOJINDO | Cat#L248 |
| **Experimental Models: Organisms/Strains** |  |  |
| C57BL/6J mice | Charles River Laboratories | https://www.criver.com/ |
| **Oligonucleotides** |  |  |
| Primers for siRNA, see Table S2 |  |  |
| Primers for ChIP-qPCR, see Table S2 |  |  |
| Primers for qPCR, see Table S2 |  |  |
| **Software and Algorithms** |  |  |
| FlowJo Software | Three Star | https://www.flowjo.com/ |
| Prism 8 | GraphPad Software | https://www.graphpad.com/  scientific-software/prism/ |
| Adobe Illustrator CS6 (version 16.0.4) | Adobe | https://www.adobe.com/ |

**Supplemental Fig.s**

**
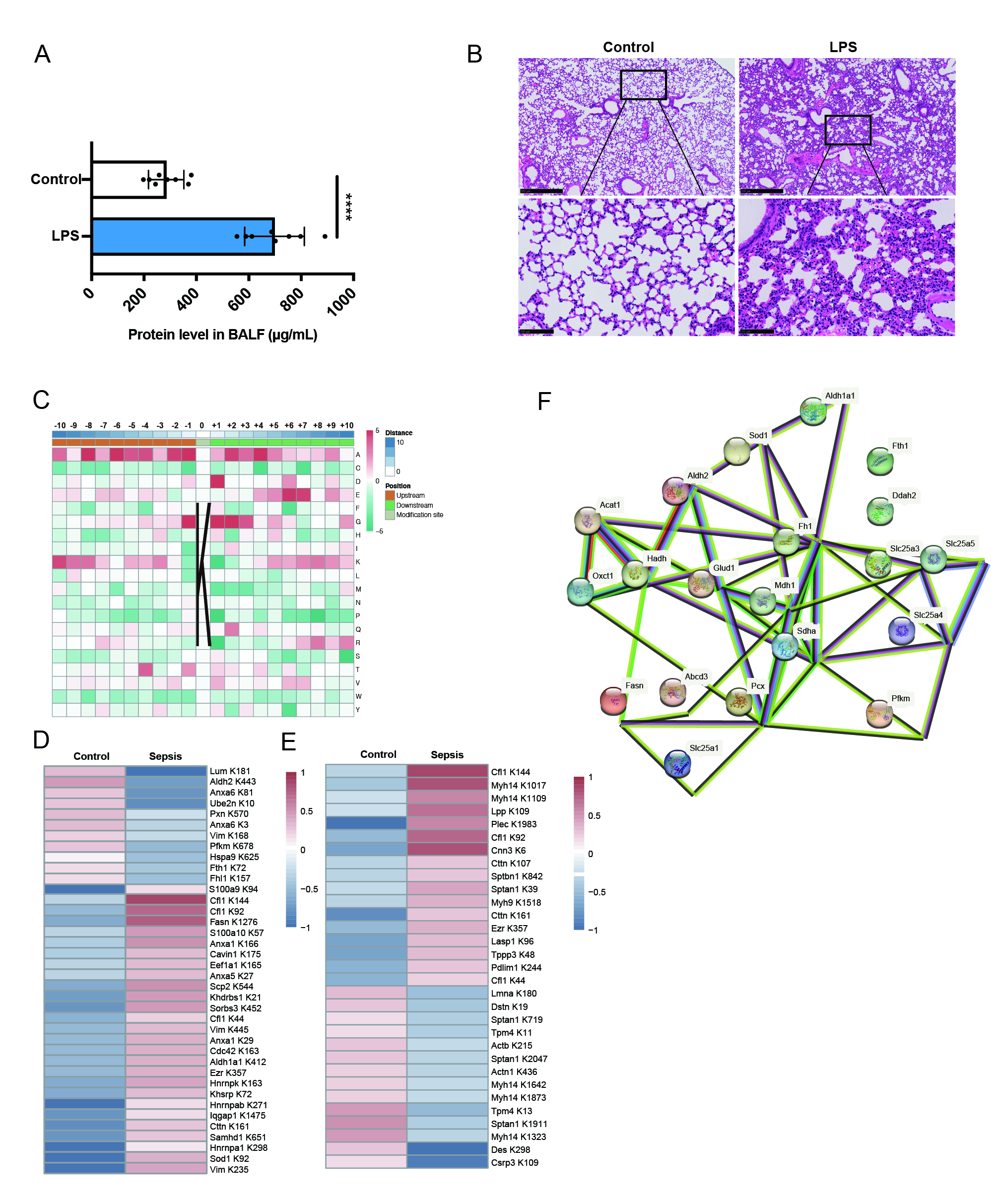
**

**Fig. S1. Enrichment analysis of lactylated proteins in lung tissues that were significantly different between septic and control mice.** (**A**) Mice were challenged with LPS (5 mg/kg) for 16h. Mice were sacrificed and the protein level in BALF was measured by BCA kit (n = 8). (**B**) The pathological injury of the lung tissues of septic mice were determined by H&E staining. (**C**) Mice were challenged with LPS (5 mg/kg) for 16h. Motif analysis of all identified lysine lactylated sites. (**D**) Heat plot of inflammation-related lactylated proteins in lung tissues between septic and control mice. (**E**) Heat plot of cytoskeleton -related lactylated proteins in lung tissues between septic and control mice. (**F**) Protein-protein interaction plot of metabolism-related lactylated proteins in lung tissues between septic and control mice.


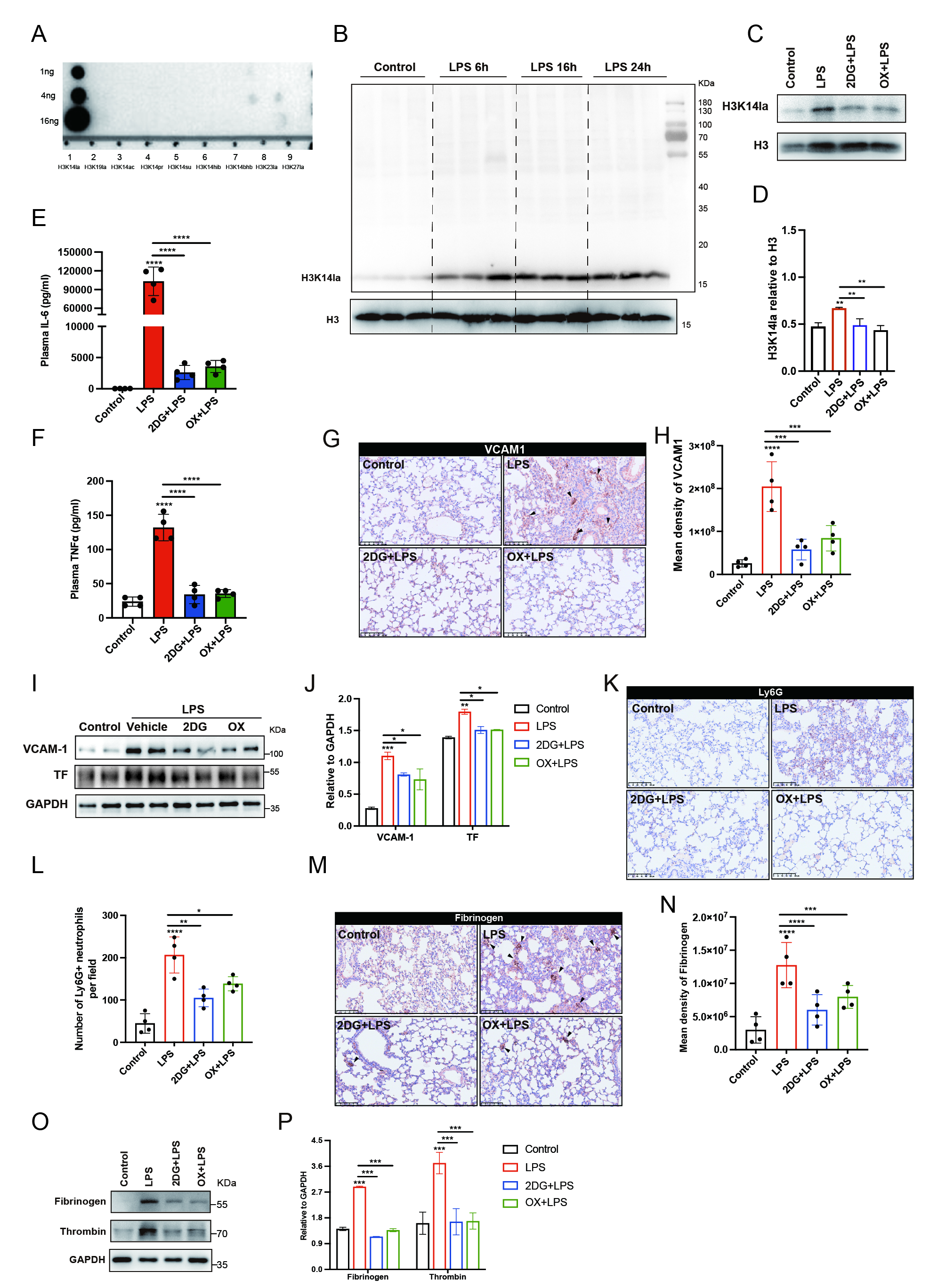


**Fig. S2. H3K14la was associated with EC activation in sepsis-induced lung injury. (A)** Dot blot assay shows the specificity of H3K14la antibody. Line1: dots contain 1, 4, 16ng H3K14la. Line 2-10: dots contain 1, 4, 16ng H3K9la, H3K14ac, H3K14pr, H3K14su, H3K14hib, H3K14bhb, H3K23la, H3K27la, H3K14un respectively. ac, acetyl lysine; bhb, β-hydroxybutyryl lysine; hib, 2-hydroxyisobutyryl lysine; la, lactyl lysine; pr, propionyl lysine; su, succinyl lysine; un, unmodified lysine. (**B**) Mice were intraperitoneally (i.p.) injected with LPS (5 mg/kg). 0.9% NaCl was i.p. injected as vehicle control. Immunoblot analysis for H3K14la in lung tissues from septic and control mice at different times. Histone H3 was taken as the loading control. (**C**, **D**) Mice were administered with 2DG (250 mg/kg) and sodium oxamate (OX, 500 mg/kg) before i.p. injection of LPS (5 mg/kg). Immunoblot of global lysine lactylation and H3K14la in lung tissues was analyzed (n=4). (**E, F**)The plasma levels of IL-6 and TNF⍺ were measured by ELISA (n = 4). (**G, H**) The expression and localization of VCAM-1 in lung tissues was detected by Immunohistochemical (IHC) staining. (**I, J**) Immunoblot analysis of the protein expressions of VCAM-1 and TF in lung tissues of mice from different groups (n=4). (**K, L**) IHC staining of neutrophil (Ly6G+) infiltration in mouse lung tissues. (**M, N**) IHC staining of fibrinogen in mouse lung tissues. (**O, P**) Immunoblot analysis of the expressions of fibrinogen and thrombin in lung tissues of mice from different groups (n=4).

**
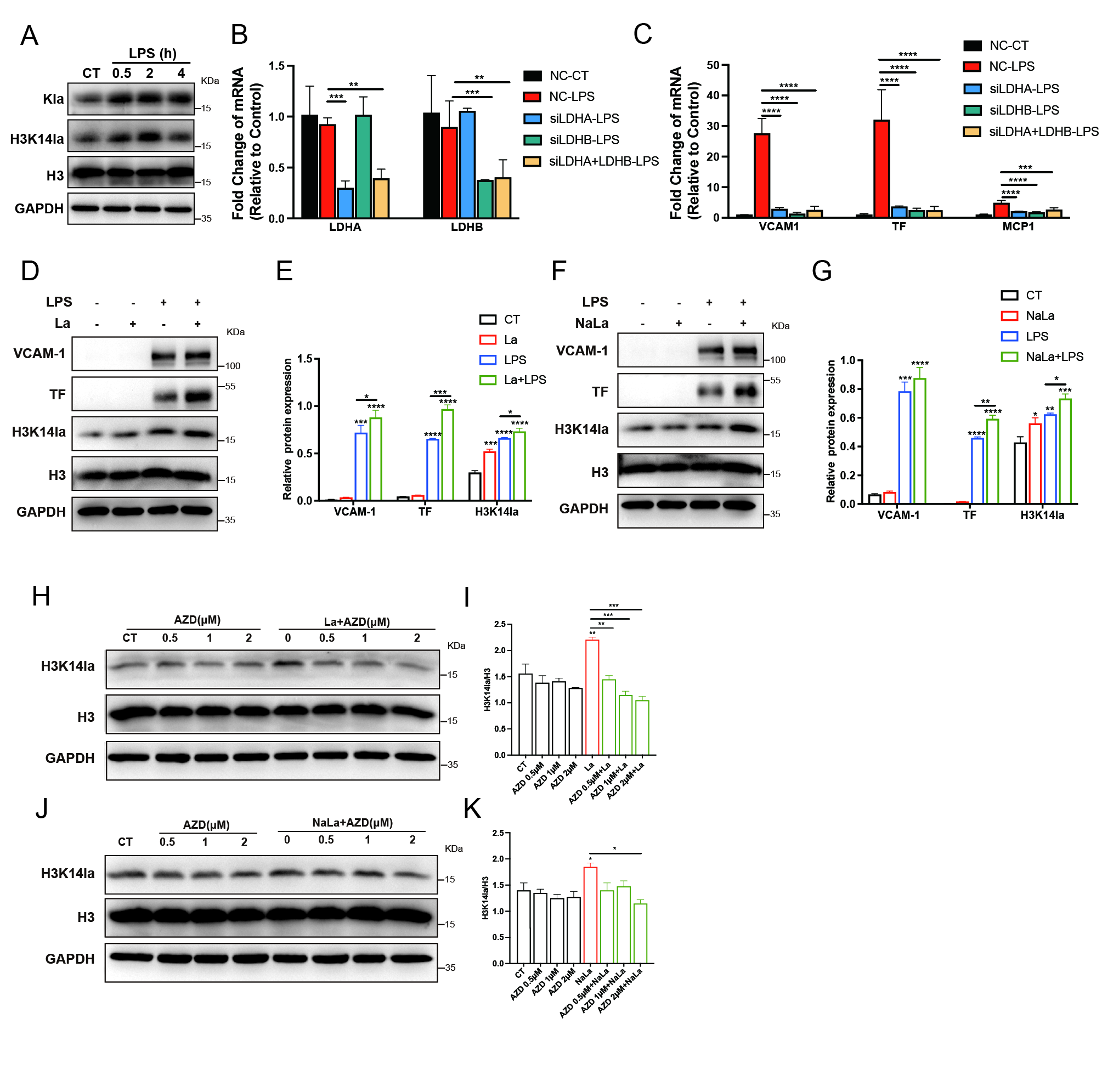
**

**Fig. S3. Lactate-dependent H3K14la levels were associated with EC activation in response to LPS*.*** (**A**) The protein levels of histone Kla and H3K14la were determined by immunoblot. (**B, C**) HUVEC were transfected with siLDHA (10nM) and siLDHB (10nM) separately or in combination. RT-qPCR analysis shows the mRNA levels of LDHA, LDHB, VCAM1, TF, and MCP1 in response to LPS in LDHA/LDHB knockdown or control HUVEC (n=3). (**D-G**) HUVEC were stimulated with lactate acid (La) and sodium lactate (NaLa), and LPS separately or in combination. The expressions of VCAM1, TF, histone Kla, and H3K14la were analyzed by western blot (n=3). (**H-K**) Immunoblot analysis shows the expressions of histone Kla and H3K14la in HUVEC treated with different concentrations of MCT inhibitor AZD3965 (AZD) before stimulated with lactate acid (La) and sodium lactate (NaLa) (n=3).


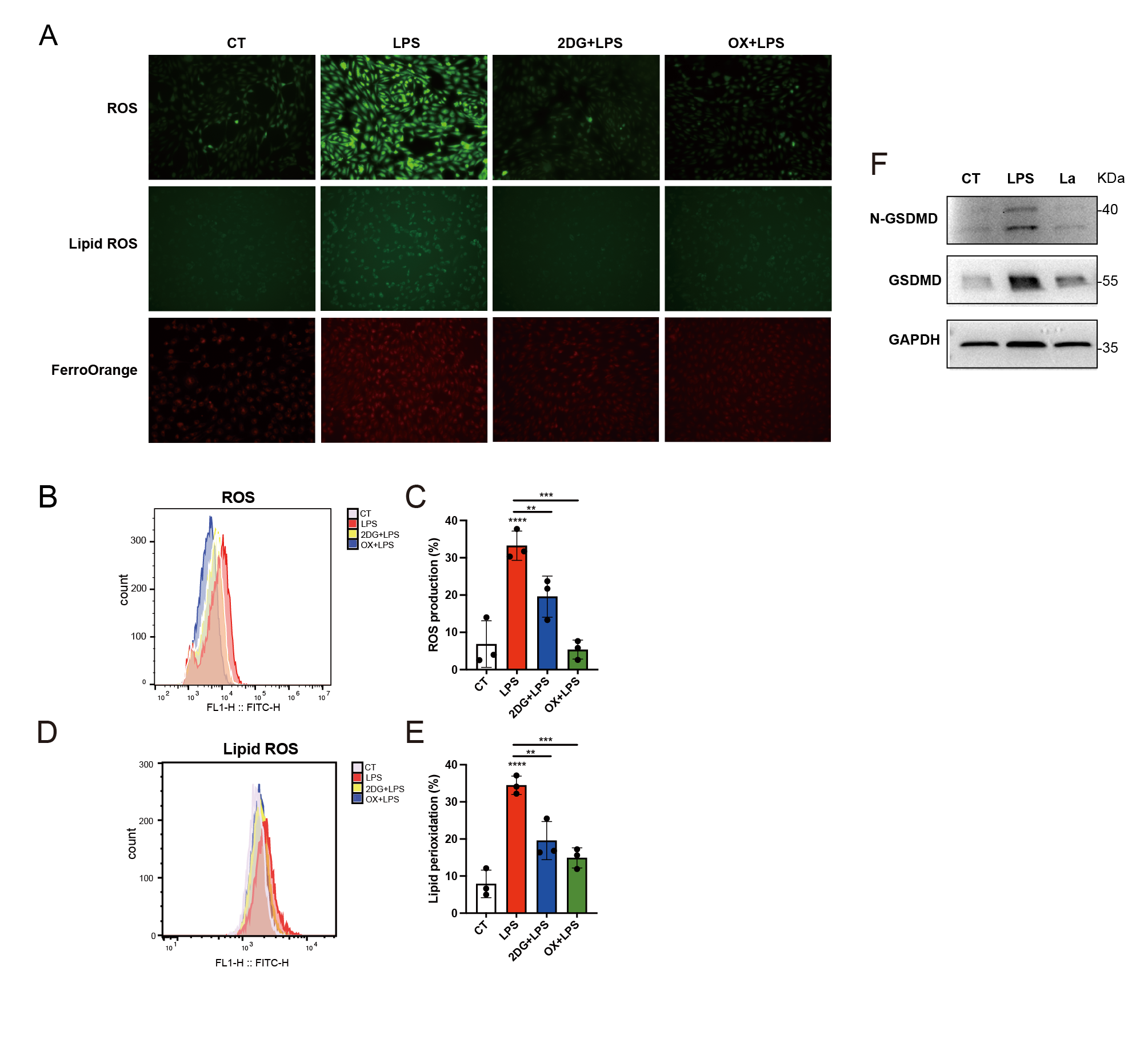


**Fig. S4. Lactate inhibition alleviated ferroptosis of HUVEC.** (**A**) HUVEC were pretreated with 2DG and sodium oxamate (OX) before LPS stimulation. The levels of ROS, lipid ROS, and FerroOrange were detected by immunofluorescent staining and orange fluorescent staining. (**B-E**) The levels of ROS and lipid ROS in HUVEC pretreated with 2DG and sodium oxamate (OX) before LPS stimulation were analyzed by flow cytometry (n = 3). (**F**) Immunoblot analysis shows the expressions of GSDMD and N-GSDMD in HUVEC stimulated with LPS and latic acid (La) separately.


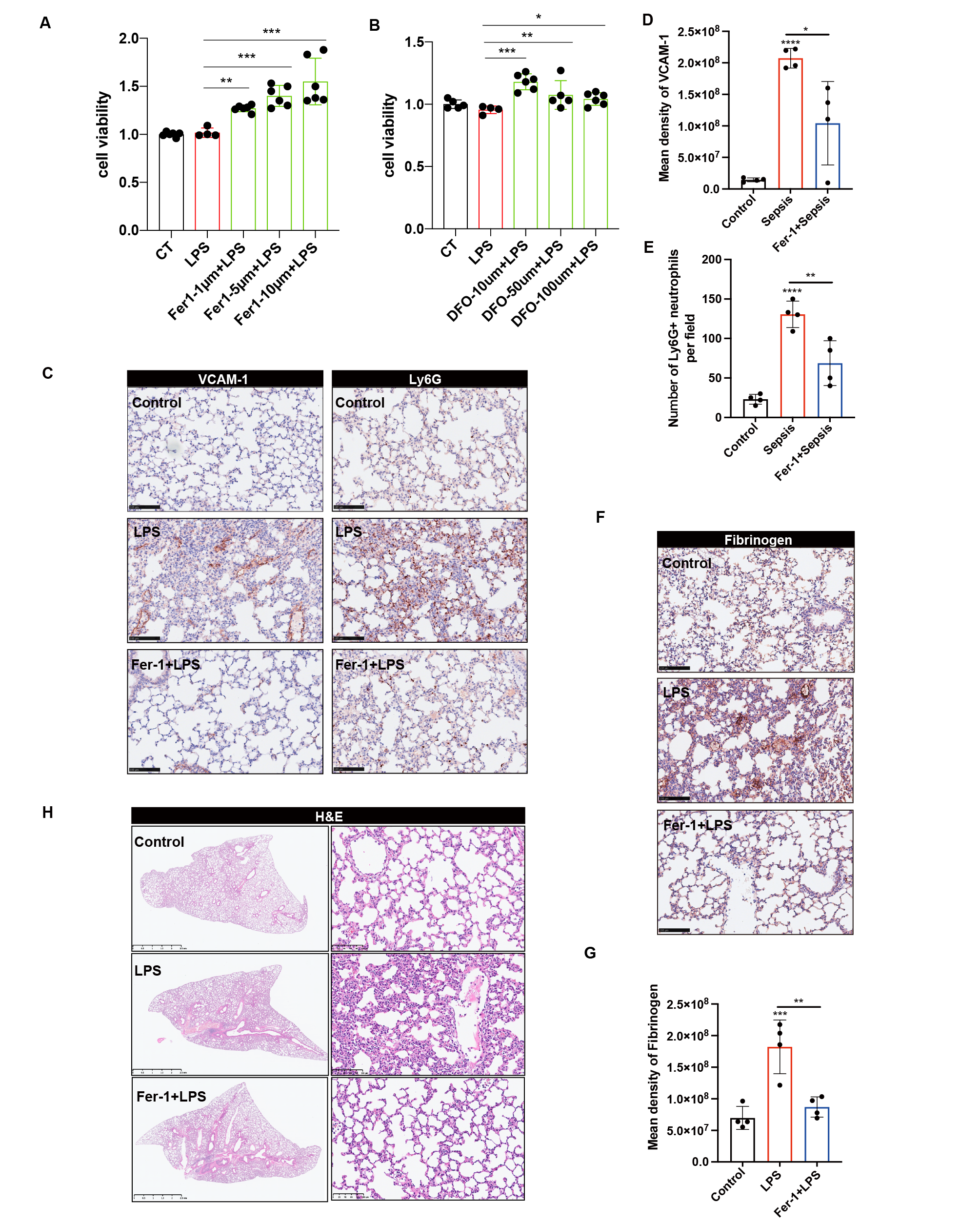


**Fig. S5. Blockade of ferroptosis alleviated endothelial dysfunction in sepsis associated lung injury.** (**A, B**) HUVEC were treated with DFO and Ferrostatin-1 before LPS stimulation. Cell survival was measured by CCK-8 assays. (**C-E**) Mice were i.p. injected with Ferrostatin-1 prior to LPS (5 mg/kg) challenge. The expression of VCAM-1 and the infiltration level of Ly6G+ neutrophils in mouse lung tissues were analyzed by IHC staining and quantified blindly by pathologists. (**F, G**) The levels of fibrinogen in the lung tissue were determined by IHC staining and quantified blindly by pathologists. (**H**) Lung tissue injury was evaluated by H&E staining.
